# Diversity and infectivity of the RNA virome among different cryptic species of an agriculturally important insect vector: whitefly *Bemisia tabaci*

**DOI:** 10.1038/s41522-021-00216-5

**Published:** 2021-05-13

**Authors:** Hai-Jian Huang, Zhuang-Xin Ye, Xin Wang, Xiao-Tian Yan, Yan Zhang, Yu-Juan He, Yu-Hua Qi, Xiao-Di Zhang, Ji-Chong Zhuo, Gang Lu, Jia-Bao Lu, Qian-Zhuo Mao, Zong-Tao Sun, Fei Yan, Jian-Ping Chen, Chuan-Xi Zhang, Jun-Min Li

**Affiliations:** State Key Laboratory for Managing Biotic and Chemical Threats to the Quality and Safety of Agro-products, Key Laboratory of Biotechnology in Plant Protection of Ministry of Agriculture and Zhejiang Province, Institute of Plant Virology, Ningbo University, Ningbo, China

**Keywords:** Microbiome, Metagenomics

## Abstract

A large number of insect-specific viruses (ISVs) have recently been discovered, mostly from hematophagous insect vectors because of their medical importance, but little attention has been paid to important plant virus vectors such as the whitefly *Bemisia tabaci*, which exists as a complex of cryptic species. Public SRA datasets of *B. tabaci* and newly generated transcriptomes of three Chinese populations are here comprehensively investigated to characterize the whitefly viromes of different cryptic species. Twenty novel ISVs were confidently identified, mostly associated with a particular cryptic species while different cryptic species harbored one or more core ISVs. Microinjection experiments showed that some ISVs might cross-infect between the two invasive whitefly cryptic species, Middle East Asia Minor 1 (MEAM1) and Mediterranean (MED), but others appeared to have a more restricted host range, reflecting the possibility of distinct long-term coevolution of these ISVs and whitefly hosts. Moreover, analysis of the profiles of virus-derived small-interfering RNAs indicated that some of the ISVs can successfully replicate in whitefly and the antiviral RNAi pathway of *B. tabaci* is actively involved in response to ISV infections. Our study provides a comprehensive analysis of the RNA virome, the distinct relationships and cross-cryptic species infectivity of ISVs in an agriculturally important insect vector.

## Introduction

Viruses are the most abundant microbes on earth, and most of those previously discovered and studied viruses are pathogens causing diseases in their plant/animal hosts^1^. Over the past decade, the development of metagenomics next-generation sequencing (mNGS) has led to the discovery of a large number of novel RNA viruses, mostly from arthropod insects. These viruses, known as insect-specific viruses (ISVs), are confined exclusively to insects and are unable to replicate in vertebrates or vertebrates cells^2^. The majority of ISVs are believed to have close relationship within their host insect^3^. Most of the ISVs detected are members of particular virus taxa, including *Baculoviridae*, *Parvoviridae*, *Flaviviridae*, *Ascoviridae*, *Togaviridae*, *Bunyavirales* (formerly *Bunyaviridae*), *Rhabdoviridae*, and a novel group described as negeviruses^4,5^. Accumulating evidence has suggested that ISVs might be ancestors of arthropod-borne viruses (arboviruses) and that the presence of ISVs may influence the physiology of the host insect as well as its competence as a vector of arboviruses^6–8^. A number of ISVs have been discovered in hematophagous insects such as mosquitoes, ticks, and fleas due to their medical importance. Several ISVs have recently been reported in some important plant virus vectors: an iflavirus in a planthopper^9^, nege/kita-like viruses in aphids^10^, and a reovirus in a leafhopper^11^. However, considering their abundance in hematophagous insects, there has been comparatively little investigation into the diversity of ISVs in plant virus vectors^12^.

With the aid of this unbiased mNGS technology, a diverse assemblage of novel viruses were revealed in various hosts, including insects^1,13^. The discovered viruses are often shared among phylogenetically related host species, perhaps because of their similar ecology and food sources, as well as selective pressures from host immune response and microbial interactions^14–16^. A comparative analysis of the virome in mosquitoes indicated that the majority of the identified viruses were mosquito species specific, and that both *Aedes aegypti* and *Culex quinquefasciatus* were associated with a number of stable eukaryotic viruses, respectively^17^. In addition, a recent virome investigation in waterfowl and shorebird communities identified both multi-host generalist viruses as well as those that appear to be host-specific, demonstrating the importance of using multi-host, multi-virus systems in the study of virus ecology^18^.

The whitefly *Bemisia tabaci* (Hemiptera: *Aleyrodidae*) causes substantial economic losses worldwide and poses a serious threat to global food security through direct feeding, excreting honeydew that promotes sooty mold, and more importantly, transmitting devastating pathogenic plant viruses^19,20^. In particular, ssDNA viruses belonging to the genus Begomovirus (*Geminiviridae*) are exclusively transmitted by *B. tabaci* in a persistent-circulative manner^21^. *B. tabaci* is a complex of morphologically indistinguishable cryptic species, and the threshold to define these cryptic species is 3.5% nucleotide divergence in their mitochondrial cytochrome oxidase subunit I (mtCOI) gene sequences^22–24^. Of these cryptic species, Middle East Asia Minor 1 (MEAM1; previously biotype B) and Mediterranean (MED; previously biotype Q) have the most important commercial impact due to their ability to spread globally, replace native whiteflies, and transmit economically important plant viruses^20,25^. Several studies suggest that many begomoviruses are transmitted by various *B. tabaci* cryptic species with different efficiencies, as for instance the differential efficiency of transmission of tomato yellow leaf curl virus and tomato yellow leaf curl Sardinia virus by MEAM1 and MED^24,26^.

Despite the increasing number of ISVs discovered in arthropod insects using mNGS, as far as we know, the diversity complex of ISVs in insect pests of agricultural importance has not yet been investigated. In this study, reassembly and extensive analysis of RNA viromes was performed with publicly available datasets (NCBI Sequence Read Archive, NCBI SRA) of *B. tabaci* distributed globally, as well as with transcriptomes generated as part of this work. Our results reveal the presence of 32 previously unreported RNA viruses in different cryptic species of *B. tabaci*. Analysis of sRNA profiles suggests that siRNA-based antiviral immunity is actively involved in the response of whiteflies to most of the ISVs. Comparative analysis and further experimental study confirmed that some ISVs might be specific to a particular cryptic species of whitefly whereas others may have a broader host range, highlighting the complex long-term coevolution between the different whitefly cryptic species and the ISVs that infect them.

## Results

### Transcriptome assembly and assignment of whitefly cryptic species

The 41 selected datasets of *B. tabaci* from the SRA repository were reassembled and the N50 of each library (assembled with Trinity) is listed in Table 1. These datasets were submitted by labs from seven countries in Asia, Europe, and America for various experimental purposes (Supplementary Fig. 1 and Table 1). The cryptic species of each assembled dataset was determined by homology search against the mtCOI database. The results indicated that the majority of the whitefly datasets belong to the invasive cryptic species MEAM1or MED, and the other whitefly cryptic species were identified include sub-Saharan Africa 1 (SSA1), New World 1 (NW1), AsiaII1, and AsiaII7 (Table 1). Meanwhile, the whitefly transcriptomes of NBU-B, NBU-Q (lab cultures) and FY-Q (field sample) were also sequenced, assembled, and assigned to the cryptic species as listed in Table 1.

**Table 1:** Whitefly datasets used in this study derived from public database, lab cultures, and field investigation.

Library	BioProject accession	Run accession number	Cryptic species ideification	University/Institute	Location	Brief description	Total base (Gb)	N50
ZJU-B1	PRJNA407873	SRR6117406	MEAM1	Zhejiang University	Hangzhou, China	Healthy whitefly (gut)	7.7	2690
ZJU-B2		SRR6117407	MEAM1			TYLCV infected whitefly (gut)	7.7	2844
ZJU-B3	PRJNA282153	SRR2001504	MEAM1			Whitefly nymph	4.7	1076
ZJU-B4	PRJNA255986	SRR1523522	MEAM1			Whitefly adult	4.7	1462
ZJU-Q1	PRJNA79601	SRR1104130	MED			Whitefly adult	10.4	2009
ZJU-Q2	PRJNA338731	SRR4039449	MED			Whitefly fed on cotton and tobacco	18.6	1111
CAAS-B1	PRJNA298415	SRR4293755	MEAM1	Chinese Academy of Agricultural Sciences	Beijing, China	Whitefly egg	7.1	2942
CAAS-B2		SRR4293724	MEAM1			Whitefly male adult	4.1	1557
CAAS-B3		SRR4293725	MEAM1			Whitefly female adult	5.1	2142
CAAS-Q1		SRR4293748	MED			Whitefly male adult	5.6	1474
CAAS-Q2		SRR2619082	MED			Unknown	5.6	1475
CAAS-BQ		SRR4293752	MEAM1 and MED			Whitefly female adult	5.5	1779
CAAS-B4	PRJEB17859	ERR1726444	MEAM1			Imidacloprid treatment	6.3	2508
CAAS-Q3		ERR1726458	MED			Imidacloprid treatment	5.5	3085
CAAS-B5	PRJNA344376	SRR4426118	MEAM1			Whitefly fed on cotton	6.2	648
CAAS-Q4		SRR4426099	MED			Whitefly fed on cotton	5.5	2308
CAAS-B6	PRJNA89143	SRR453543	MEAM1			Different plant host and whitefly sex strains	5.2	1114
CAAS-Q5	PRJNA276952	SRR2895294	MED			Sexual differences of whitefly	4.1	1166
CAAS-B7	PRJNA391229	SRR5723126	MEAM1			Whitefly antenna	5.0	1186
CAU-Q1	PRJNA417353	SRR6262725	MED	China Agricultural University	Beijing, China	Wolbachia related study	8.3	1128
CAS-Q1	PRJNA606896	SRR11092386	MED	Chinese Academy of Sciences	Beijing, China	Whitefly head	6.2	2083
QAU-BQ	PRJNA490883	SRR7829909	MEAM1 and MED	Qingdao Agricultural University	Qingdao, China	Whiteflies co-infected by TYLCV and ToCV	5.2	2055
QAU-Q1	PRJNA279224	SRR1930109	MED			Interaction between whitefly and plant hosts	5.0	2141
NIAS-Q1	PRJDB2008	DRR018506	MED	National Institute of Agrobiological Sciences	Tsukuba, Japan	Study of mitochondrial transporters	3.4	2430
NABI-A1	PRJNA237273	SRR1159208	AsiaII7	National Agri-Food Biotechnology Institute	Ajitgarh, India	Comprehensive transcriptome analysis	8.5	2237
UE-Q1	PRJEB13160	ERR1337902, ERR1337901, ERR1337900	MED	University of Exeter	Penryn, UK	Study of insecticide resistance	11.4	2851
UC-Q1	PRJNA293094	SRR2174325	MED	University of Crete	Heraklion, Greece	Study of insecticide resistance	7.8	1877
CU-B1	PRJNA312467	SRR3179979	MEAM1	Cornell University	New York, USA	Fed on tomato	4.2	1339
CU-S1	PRJNA419386	SRR6313815	SSA1			Whitefly adults	7.4	597
HU-Q1	PRJNA427517	SRR6432772	MED—France	The Hebrew University	Jerusalem, Israel	Differential expression in whitefly species	2.2	1036
HU-Q2		SRR6432775	MED—France				2.1	989
HU-Q3		SRR6432776	MED—France				2.0	878
HU-S1		SRR6432785	SSA—Tanzania				2.2	1560
HU-S2		SRR6432829	SSA1—Tanzania				2.1	853
HU-S3		SRR6432827	SSA1—Tanzania				2.1	846
HU-A1		SRR6432767	AsiaII1				2.1	667
HU-A2		SRR6432762	AsiaII1				2.1	1088
HU-A3		SRR6432768	AsiaII1				2.1	736
HU-N1		SRR6432809	NW1—Brazil				2.3	1367
HU-N2		SRR6432804	NW1—Brazil				2.0	1152
HU-N3		SRR6432800	NW1—Brzil				1.9	1425
NBU-B	PRJNA677841	SRR13050950	MEAM1	Ningbo University	Ningbo, China	Lab culture of this study	14	2902
NBU-Q		SRR13052369	MED				15	3385
FY-Q		SRR13039280	MED		Fuyang, China	Field investigation	13	2676

*MEAM1* Middle East Asia Minor 1, *MED* Mediterranean, *SSA1* sub-Saharan Africa 1, *NW1* New World 1.

### Diversity of RNA viromes discovered in *B. tabaci*

**Fig. 1:**
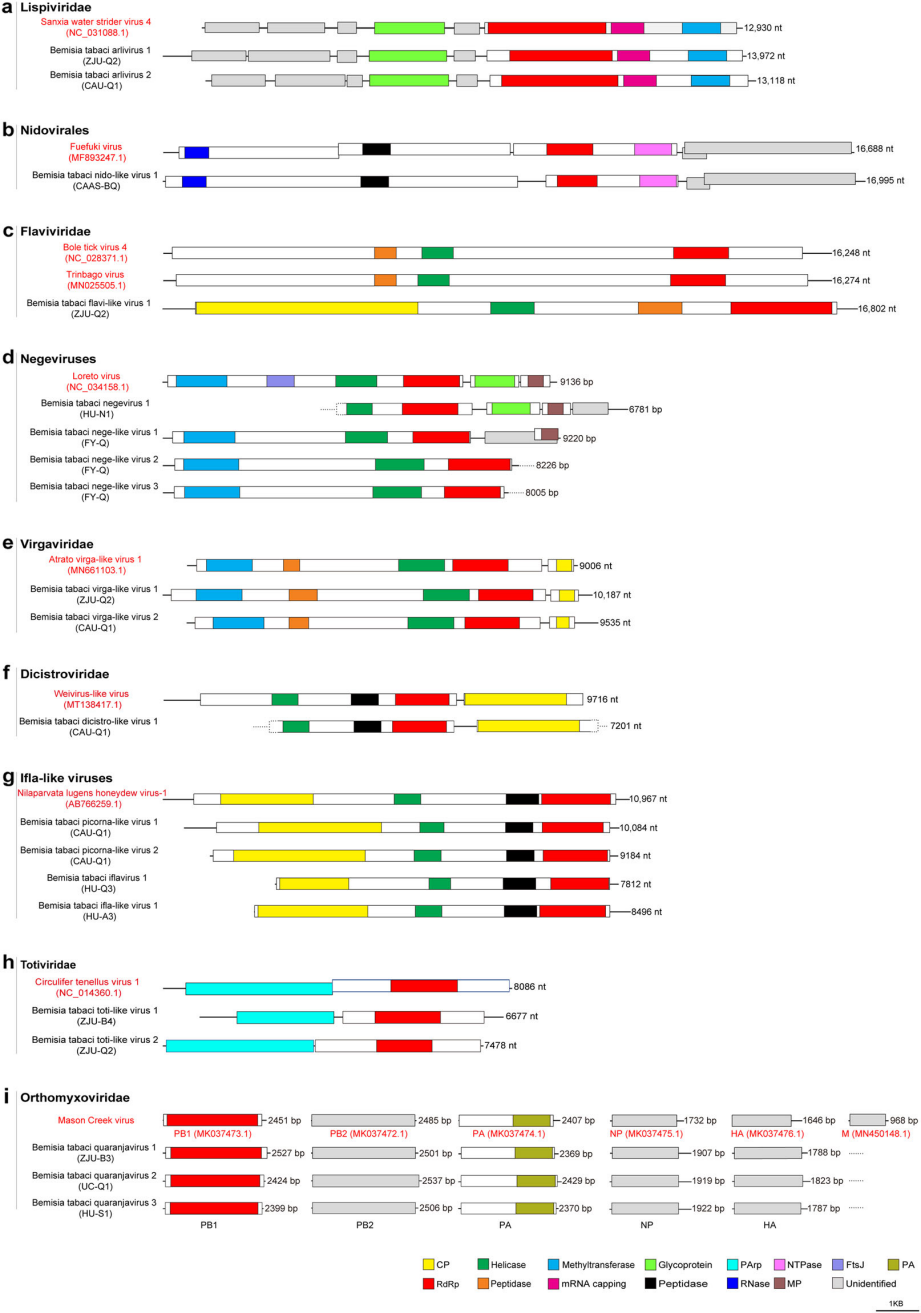
Genomic structures of novel insect-specific viruses identified in whitefly *B. tabaci*. The viruses were taxonomically classified into nine groups as shown in panels **a**–**i**. Each panel contains a genome representing a phylogenetically close reference virus on the top (with red font) and the insect-specific viruses discovered from whiteflies in this study. GenBank accession numbers are provided in parentheses after the name of the reference viruses. The name of the whitefly dataset corresponding to the identified novel viruses is also indicated in parentheses below the virus name (details in Table 2). Conserved functional domains are color-coded and the names of the domains are indicated at the bottom of the figure. Abbreviation of the conserved domain names: *CP* coat protein, *FtsJ* RNA ribosomal methyltransferase, *MP* membrane protein, *PA* polymerase, *PArp* proline–alanine-rich protein, *RdRp* RNA-dependent RNA polymerase.

**Fig. 2:**
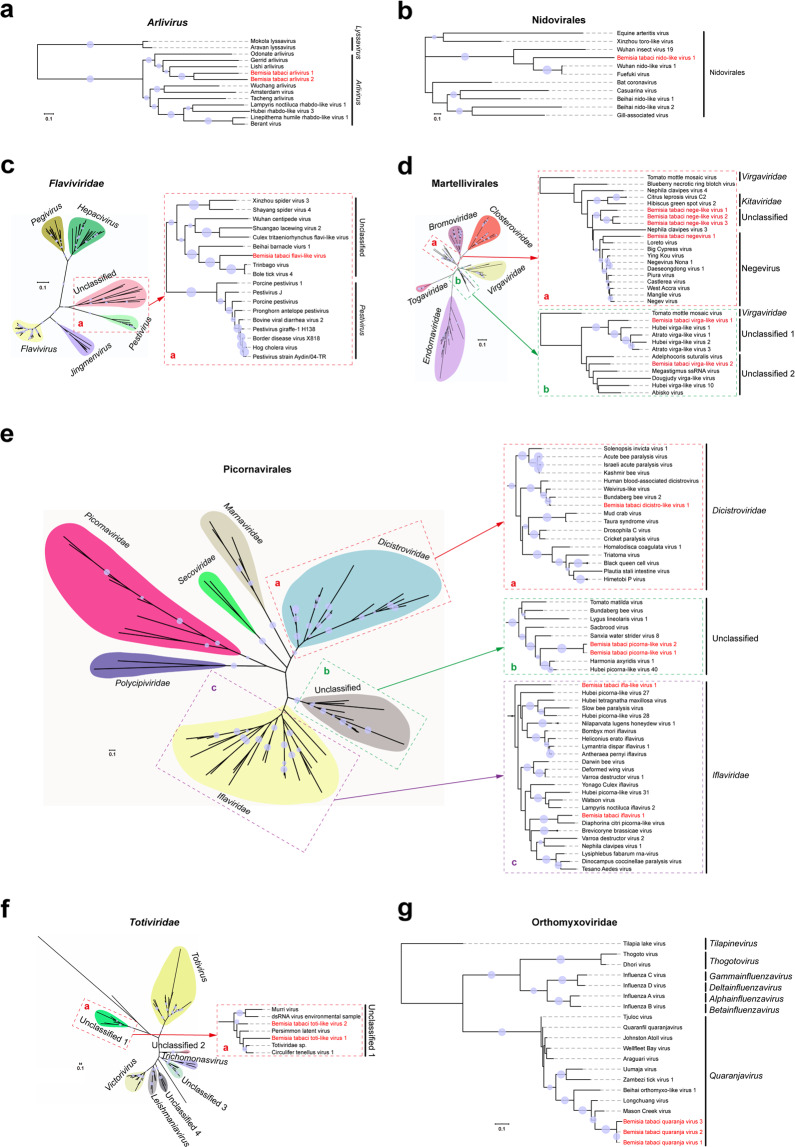
Phylogeny of novel insect-specific viruses (ISVs) identified in whitefly *B. tabaci* with other related viruses. Trees for *Arlivirus* (**a**), Nidovirales (**b**), *Flaviviridae* (**c**), Martellivirales (**d**), Picornavirales (**e**), *Totiviridae* (**f**), and *Orthomyxoviridae* (**g**) are based on the maximum likelihood method and inferred from conserved viral RdRp domains. Novel ISVs identified in this study are shown in red font. Nodes with bootstrap values >50% are marked with solid blue circles, and the larger circles indicate higher bootstrap values. In panels **c**–**f**, a taxonomic overview of viruses at order or family level are shown on the left, and a close-up view of the viruses of interest in this study are shown in the dotted frames on the right. The viral sequences used in this study were extracted from GenBank: the accession numbers and other related details are listed in Supplementary Table 2.

Across all of the assembled libraries and using the strict criteria described above, 32 novel RNA viruses (20 ISVs and 12 plant/fungal virus-like contigs) (Table 2), and a new isolate of Potato virus S (PVS) were identified in *B. tabaci*. Based on the taxonomy of the most closely related reference viruses, the 20 newly discovered RNA viruses were putative members of or related to the following orders/families: *Lispiviridae* (*N* = 2), Nidovirales (*N* = 1), *Flaviviridae* (*N* = 1), Negevirus (newly proposed taxon, *N* = 4), *Virgaviridae* (*N* = 2), Picornavirales (*N* = 5), *Orthomyxoviridae* (*N* = 3), and *Totiviridae* (*N* = 2). The genomic structures and phylogeny of these ISVs, together with related reference viruses, are shown in Figs.1 and 2. Two novel negative-sense single-stranded RNA (ssRNA) viral genomes were identified, and clearly cluster together with other viruses in the genus Arlivirus, which mainly infect invertebrates such as insects, spiders, and nematodes (Figs. 1a and 2a). Positive-sense ssRNA virus discovered in whitefly including a nido-like virus (Figs. 1b and 2b), a flavi-like virus (Figs. 1c and 2c), a negevirus and three nege-like viruses (Figs. 1d and 2d), two virga-like viruses (Figs. 1e and 2d), a dicistro-like virus (Figs. 1f and 2e), and four ifla-like viruses (Figs. 1g and 2e). Furthermore, we also detected two toti-like dsRNA viruses (Figs. 1h and 2f), and three closely related segmented negative ssRNA quaranjaviruses (Figs. 1i and 2g and Supplementary Fig. 2) in *B. tabaci*, suggesting the high diversity of ISVs in whiteflies. Besides the 20 novel ISVs described above, we also identified 12 diverse plant/fungal virus-like contigs in the whiteflies collected from field (sample FY-Q), and a new isolate of PVS in the whitefly dataset CU-B1 (Supplementary Fig. 3). Dataset FY-Q contained a total of six beny-like viruses and six bromo-like viruses with relatively high abundance (coverage) (Table 2 and Supplementary Fig. 4). Detailed descriptions for each of the identified novel virus are provided in Supplementary Result 1. In addition, tissue expression analysis of ISVs by quantitative reverse transcription PCR (qRT-PCR) showed that Bemisia tabaci quaranjavirus 1 (BtQuV1) was ubiquitously expressed in all tissues of NBU-B, whereas Bemisia tabaci virga-like virus 2 (BtViLV2) was mostly accumulated in the gut of NBU-Q (Supplementary Fig. 5).

**Table 2:** Novel viruses identified in whiteflies from publicly available databases and field samples.

Tentative virus names	NCBI accession	Library	Length (nt)	Coverage	*E*-value	Homologous virus (genome size, nt)	Protein identities	Virus family	Virus genus	Homologous virus reference
Bemisia tabaci arlivirus 1 (BtArV1)	MW256666	ZJU-Q2	13,972	965×	0.0	Lishi Spider Virus 2 (9924)	30%	*Lispiviridae*	Arlivirus	^60^
Bemisia tabaci arlivirus 2 (BtArV2)	MW256667	CAU-Q1	13,118	250×	0.0	Hubei odonate virus 10 (14,440)	28%	*Lispiviridae*	Arlivirus	^61^
Bemisia tabaci nido-like virus 1 (BtNiLV1)	MW256673	CAAS-BQ	16,995	414×	4e−157	Wuhan insect virus 19 (15,441)	33%	Unassigned	Unassigned	^61^
Bemisia tabaci flavi-like virus 1 (BtFlLV1)	MW256672	ZJU-Q2	16,802	22×	2e−161	Bole tick virus 4 (16,248)	34%	*Flaviviridae*	Unassigned	^62^
Bemisia tabaci negevirus 1 (BtNeV1)	MW256675	HU-N1	6781	72×	0.0	Loreto virus (9136)	41%	Unassigned	Unassigned	^63^
Bemisia tabaci nege-like virus 1 (BtNeLV1)	MW256676	FY-Q	9220	163×	3e−174	Wuhan house centipede virus 1 (10,310)	36%	Unassigned	Unassigned	^64^
Bemisia tabaci nege-like virus 2 (BtNeLV2)	MW256677	FY-Q	8226	46×	4e−170	Big Cypress virus (9506)	36%	Unassigned	Unassigned	^65^
Bemisia tabaci nege-like virus 3 (BtNeLV3)	MW256678	FY-Q	8005	58×	4e−171	Big Cypress virus (9506)	35%	Unassigned	Unassigned	^65^
Bemisia tabaci virga-like virus 1 (BtViLV1)	MW256664	ZJU-Q2	10,187	137×	0.0	Hubei virga-like virus 1 (9141)	40%	*Virgaviridae*	Unassigned	^61^
Bemisia tabaci virga-like virus 2 (BtViLV2)	MW256665	CAU-Q1	9535	3297×	3e−172	Megastigmus ssRNA virus (10,187)	35%	*Virgaviridae*	Unassigned	^66^
Bemisia tabaci dicistro-like virus 1 (BtDiLV1)	MW256674	CAU-Q1	7201	125×	0.0	Bundaberg bee virus 2 (7955)	84%	*Dicistroviridae*	Unassigned	^67^
Bemisia tabaci picorna-like virus 1 (BtPiLV1)	MW256668	CAU-Q1	10,084	86×	1e−123	Hubei tetragnatha maxillosa virus 2 (9763)	27%	Unassigned	Unassigned	^61^
Bemisia tabaci picorna-like virus 2 (BtPiLV2)	MW256669	CAU-Q1	9184	50×	3e−137	Sanxia water strider virus 8 (9166)	38%	Unassigned	Unassigned	^61^
Bemisia tabaci iflavirus 1 (BtIfV1)	MW256671	HU-Q3	7812	78×	1e−165	Nephila clavipes virus 1 (10,198)	29%	*Iflaviridae*	*Iflavirus*	^68^
Bemisia tabaci ifla-like virus 1 (BtIfLV1)	MW256670	HU-A3	8496	44×	1e−142	Sanxia water strider virus 8 (9166)	36%	*Iflaviridae*	Unassigned	^61^
Bemisia tabaci toti-like virus 1 (BtToLV1)	MW227222	ZJU-B4	6677	26×	1.9e−118	Circulifer tenellus virus 1 (8086)	34%	*Totiviridae*	Unassigned	^69^
Bemisia tabaci toti-like virus 2 (BtToLV2)	MW227223	ZJU-Q2	7478	47×	2.1e−155	Persimmon late virus (7475)	55%	*Totiviridae*	Unassigned	^70^
Bemisia tabaci Quaranjavirus 1 (BtQuV1)	MW256682	ZJU-B3	2501	111×	1e−86	Mason Creek virus (Seg. 1, PB2, 2485)	28%	*Orthomyxoviridae*	Quaranjavirus	^71^
	MW256679		2369	138×	3e−163	Mason Creek virus (Seg. 2, PA, 2407)	37%			
	MW256680		2541	177×	7e−141	Beihai orthomyxo-like virus 1 (Seg. 3, PB1, 2432)	45%			
	MW256681		1907	519×	7e−96	Mason Creek virus (Seg. 4, NP, 1732)	34%			
	MW256683		1788	274×	1e−75	Mason Creek virus (Seg. 5, HA, 1646)	29%			
Bemisia tabaci Quaranjavirus 2 (BtQuV2)	MW256687	UC-Q1	2537	69×	1e−84	Mason Creek virus (Seg. 1, PB2, 2485)	28%	*Orthomyxoviridae*	Quaranjavirus	^71^
	MW256685		2429	107×	1e−162	Mason Creek virus (Seg. 2, PA, 2407)	37%			
	MW256686		2424	223×	0.0	Mason Creek virus (Seg. 3, PB1, 2451)	56%			
	MW256688		1919	196×	1e−93	Mason Creek virus (Seg. 4, NP, 1732)	34%			
	MW256684		1823	290×	1e−73	Mason Creek virus (Seg. 5, HA, 1646)	29%			
Bemisia tabaci Quaranjavirus 3 (BtQuV3)	MW256692	HU-S1	2506	33×	3e−92	Mason Creek virus (Seg. 1, PB2, 2485)	29%	*Orthomyxoviridae*	Quaranjavirus	^71^
	MW256690		2370	63×	2e−159	Mason Creek virus (Seg. 2, PA, 2407)	37%			
	MW256691		2399	33×	0.0	Mason Creek virus (Seg. 3, PB1, 2451)	56%			
	MW256693		1922	124×	2e−79	Mason Creek virus (Seg. 4, NP, 1732)	34%			
	MW256689		1787	60×	3e−73	Mason Creek virus (Seg. 5, HA, 1646)	29%			
Bemisia tabaci beny-like virus 1 (BtBeLV1)	MW256694	FY-Q	6479	42×	0.0	Agaricus bisporus virus 8 (8280, partial)	32%	*Benyviridae*	Benyvirus	N/A
Bemisia tabaci beny-like virus 2 (BtBeLV2)	MW256695	FY-Q	6369	23×	0.0	Agaricus bisporus virus 8 (8280, partial)	32%	*Benyviridae*	Benyvirus	N/A
Bemisia tabaci beny-like virus 3 (BtBeLV3)	MW256696	FY-Q	6209	44×	0.0	Agaricus bisporus virus 8 (8280, partial)	32%	*Benyviridae*	Benyvirus	N/A
Bemisia tabaci beny-like virus 4 (BtBeLV4)	MW256697	FY-Q	6343	219×	0.0	Agaricus bisporus virus 8 (8280, partial)	31%	*Benyviridae*	Benyvirus	N/A
Bemisia tabaci beny-like virus 5 (BtBeLV5)	MW256698	FY-Q	6229	31×	0.0	Agaricus bisporus virus 8 (8280, partial)	31%	*Benyviridae*	Benyvirus	N/A
Bemisia tabaci beny-like virus 6 (BtBeLV6)	MW256699	FY-Q	5127	96×	0.0	Hubei Beny-like virus 1 (4365)	76%	*Benyviridae*	Benyvirus	^61^
Bemisia tabaci bromo-like virus 1 (BtBromoLV1)	MW256700	FY-Q	6158	181×	1e−82	Beihai charybdis crab virus 1 (6969)	38%	*Bromoviridae*	Unassigned	^61^
Bemisia tabaci bromo-like virus 2 (BtBromoLV2)	MW256701	FY-Q	6215	523×	2e−79	Beihai charybdis crab virus 1 (6969)	36%	*Bromoviridae*	Unassigned	^61^
Bemisia tabaci bromo-like virus 3 (BtBromoLV3)	MW256702	FY-Q	6005	494×	7e−82	Beihai charybdis crab virus 1 (6969)	38%	*Bromoviridae*	Unassigned	^61^
Bemisia tabaci bromo-like virus 4 (BtBromoLV4)	MW256703	FY-Q	6631	36×	5e−66	Beihai charybdis crab virus 1 (6969)	35%	*Bromoviridae*	Unassigned	^61^
Bemisia tabaci bromo-like virus 5 (BtBromoLV5)	MW256704	FY-Q	6529	126×	3e−51	Beihai charybdis crab virus 1 (6969)	35%	*Bromoviridae*	Unassigned	^61^
Bemisia tabaci bromo-like virus 6 (BtBromoLV6)	MW256705	FY-Q	5972	45×	2e−37	Beihai charybdis crab virus 1 (6969)	30%	*Bromoviridae*	Unassigned	^61^

### Abundance and association of whitefly ISVs with host cryptic species

**Fig. 3:**
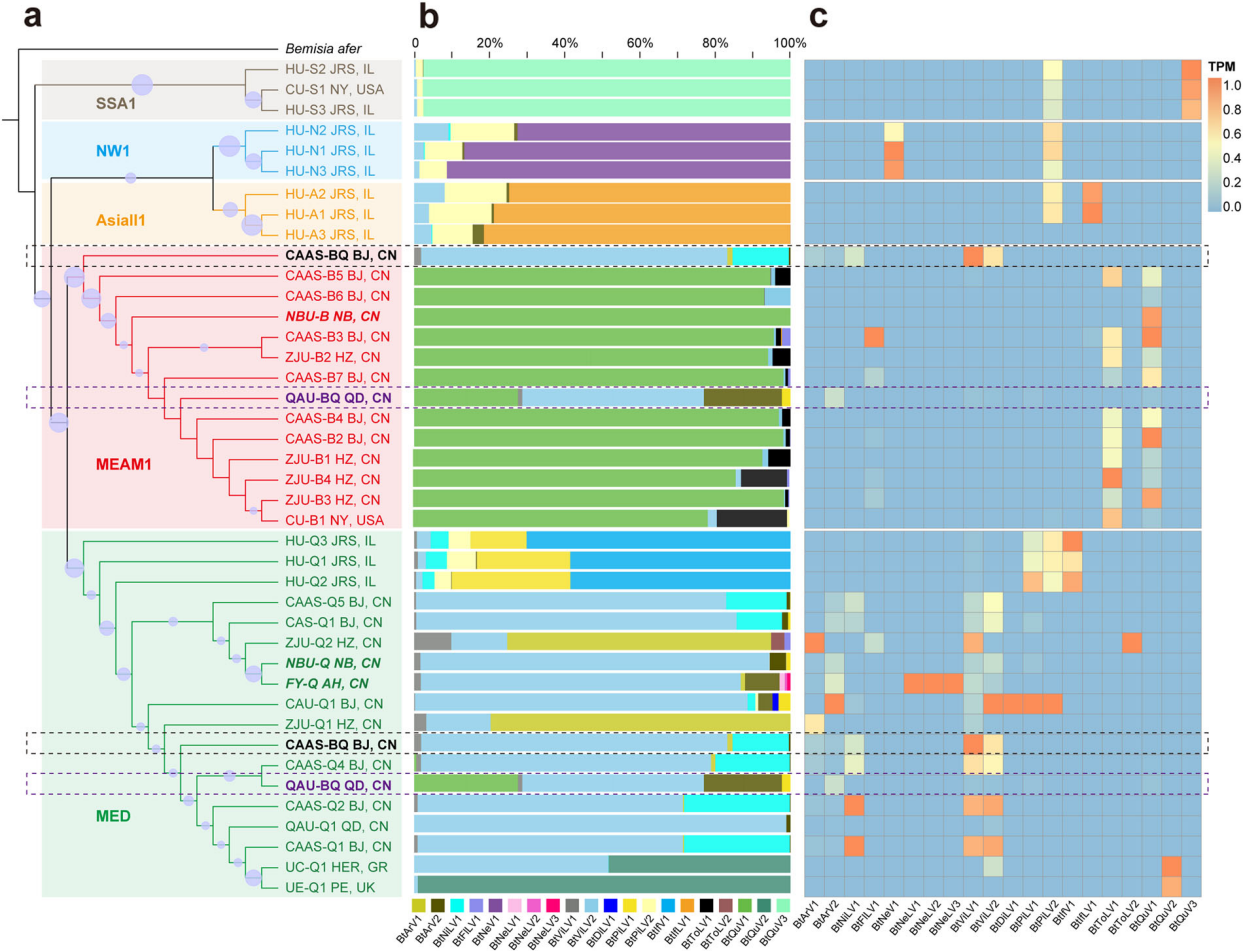
Correlation between whitefly cryptic species and insect-specific viruses. **a** Phylogeny of whitefly cryptic species based on mtCOI sequences using the maximum likelihood method. The mtCOI of *Bemisia afer* (MK360160.1) was used to root the tree. Two datasets (CAAS-BQ BJ,CN and QAU-BQ QD,CN) containing mixed cryptic species (MEAM1 and MED) are highlighted by dotted frames colored with black and purple, respectively. Nodes with bootstrap values >50% are marked with solid blue circles. **b** Composition of insect-specific viruses (ISVs) in each whitefly cryptic species dataset. ISVs are color-coded and the names of viruses are indicated at the bottom of the figure. **c** Relative abundance of ISVs across the different whitefly cryptic species datasets. The transcripts per million (TPM) of each ISV are displayed by the heat map. Abbreviations of the cities and countries: JRS, IL: Jerusalem, Israel; NY: New York, USA; AJ, IN: Ajitgarh, India; BJ, CN: Beijing, China; NB, CN: Ningbo, China; HZ, CN: Hangzhou, China; QD, CN: Qingdao, China; AH, CN: Anhui, China; HER, GR: Heraklion, Greece; PE: Penryn. Abbreviation and details of the whitefly datasets and newly discovered ISVs are listed in Tables 1 and 2, respectively. Abbreviations of the whitefly cryptic species: *MEAM1* Middle East Asia Minor 1, *MED* Mediterranean, *NW1* New World 1, *SSA1* sub-Saharan Africa 1.

To illustrate the abundance, prevalence, and association of whitefly ISVs with host cryptic species, a phylogenetic tree was reconstructed based on the whitefly mtCOI sequence and its relationship with the RNA virome of each whitefly dataset was investigated. All of the whitefly datasets separate distinctly and form as five groups, corresponding to the five cryptic species of whitefly MEAM1 (*N* = 14), MED (*N* = 18), AsiaII1 (*N* = 3), NW1 (*N* = 3), and SSA1 (*N* = 3). Two of the whitefly datasets, CAAS-BQ (SRR4293752) and QAU-BQ (SRR7829909), contain a mixture of whiteflies cryptic species from both the MEAM1 and MED clades as indicated in Fig. 3a.

Analysis of ISVs composition (percentage) indicated that most of the whitefly populations (datasets) harbor stable dominant (core) ISVs which are obviously associated with a particular cryptic species of *B. tabaci*. More than 95% of the viral reads are derived from Bemisia tabaci quaranjavirus 3 (BtQuV3) for the cryptic species of SSA1, while the dominant virus for the cryptic species NW1 and AsiaII1 are, respectively, Bemisia tabaci negevirus 1 (BtNeV1) and Bemisia tabaci ifla-like virus 1 (BtIfLV1) (Fig. 3b). Although most of the datasets of these three whitefly cryptic species were submitted by the Hebrew University (Jerusalem, Israel) with limited numbers of datasets, the high similarity of ISVs composition derived from another SSA1 dataset (CU-S1) submitted by Cornell University (New York, USA) with HU-S2 and HU-S3 strongly supports the hypothesis that there is a stable and distinct group of core viruses in each of the three whitefly cryptic species (Fig. 3b). For the whitefly clade MEAM1, the dominant ISV is apparently BtQuV1 in all of the datasets (excluding CAAS-BQ and QAU-BQ) irrespective of the whitefly origin, and Bemisia tabaci toti-like virus 1 (BtToLV1) is also prevalent in most of the MEAM1 whitefly datasets (Fig. 3b). On the other hand, the RNA virome and core viruses in MED are more diverse than in the other cryptic species and the ISVs of MED are related to the geographical location of the whitefly populations as well as the clusters in the phylogenetic tree (Fig. 3b). BtViLV2 is the most prevalent and dominant virus in most of the MED datasets from China. But the two datasets from Zhejiang University (Hangzhou, China) (ZJU-Q1 and ZJU-Q2), both have Bemisia tabaci arlivirus 1 (BtArV1) as the dominant virus. The other two MED datasets from Europe, UC-Q1 (Heraklion, Greece) and UE-Q1 (Penryn, UK), group together and harbor another specific core virus Bemisia tabaci quaranjavirus 2 (BtQuV2), whereas the three MED datasets from Hebrew University (Jerusalem, Israel) also contain distinctly different viral composition including Bemisia tabaci iflavirus 1 (BtIfV1), Bemisia tabaci picorna-like virus 1 (BtPiLV1), and Bemisia tabaci picorna-like virus 2 (BtPiLV2) (Fig. 3b). Principal component analysis (PCA) also confirms the relationship between the core viruses and the various cryptic species (Supplementary Fig. 6). It is worth mentioning that in the datasets with mixed cryptic species, CAAS-BQ has a similar RNA virome composition to the other MED datasets, whereas QAU-BQ clearly has the combined core viruses of both MEM1 (BtQuV1) and MED (BtViLV2) (Fig. 3b).

The heat map shown in Fig. 3c consolidates the above observation that the RNA virome diversity is more complex in MED than in the other four cryptic species. Most of the whitefly ISVs (*N* = 13) appeared to be MED-specific, while some of the other ISVs are present specifically in MEAM1 (BtToLV1 and BtQuV1), NW1 (BtNeV1), AsiaII1 (BtIfLV1), or SSA1 (BtQuV3). Bemisia tabaci flavi-like virus 1 (BtFlLV1) is present in the datasets of both MEAM1 (*N* = 5) and MED (*N* = 1), whereas BtPiLV2 is present in most datasets of the four whitefly cryptic species from Hebrew University (Jerusalem, Israel) (Fig. 3c), implying that BtPiLV2 may have a broader host range amongst the different whitefly cryptic species. It is also interesting that Bemisia tabaci toti-like virus 2 (BtToLV2) and Bemisia tabaci dicistro-like virus 1 (BtDiLV1) are only present in datasets ZJU-Q2 and CAU-Q1, respectively, and the three nege-like viruses of *B. tabaci* (BtNeLV1-3) were exclusively discovered in the field sample FY-Q (Fig. 3c). In addition, the three phylogenetically related quaranjaviruses (BtQuV1-3) (Fig. 2g) were found in three cryptic species of whitefly MEAM1, MED, and SSA1, respectively. BtQuV1 and BtQuV3 were identified in each dataset of their respective cryptic species, whereas BtQuV2 was only found in two MED whitefly datasets originating from Europe (Fig. 3c).

### Experimental evaluation of the ability of whitefly ISVs to cross-infect the cryptic species MEAM1 and MED

**Fig. 4:**
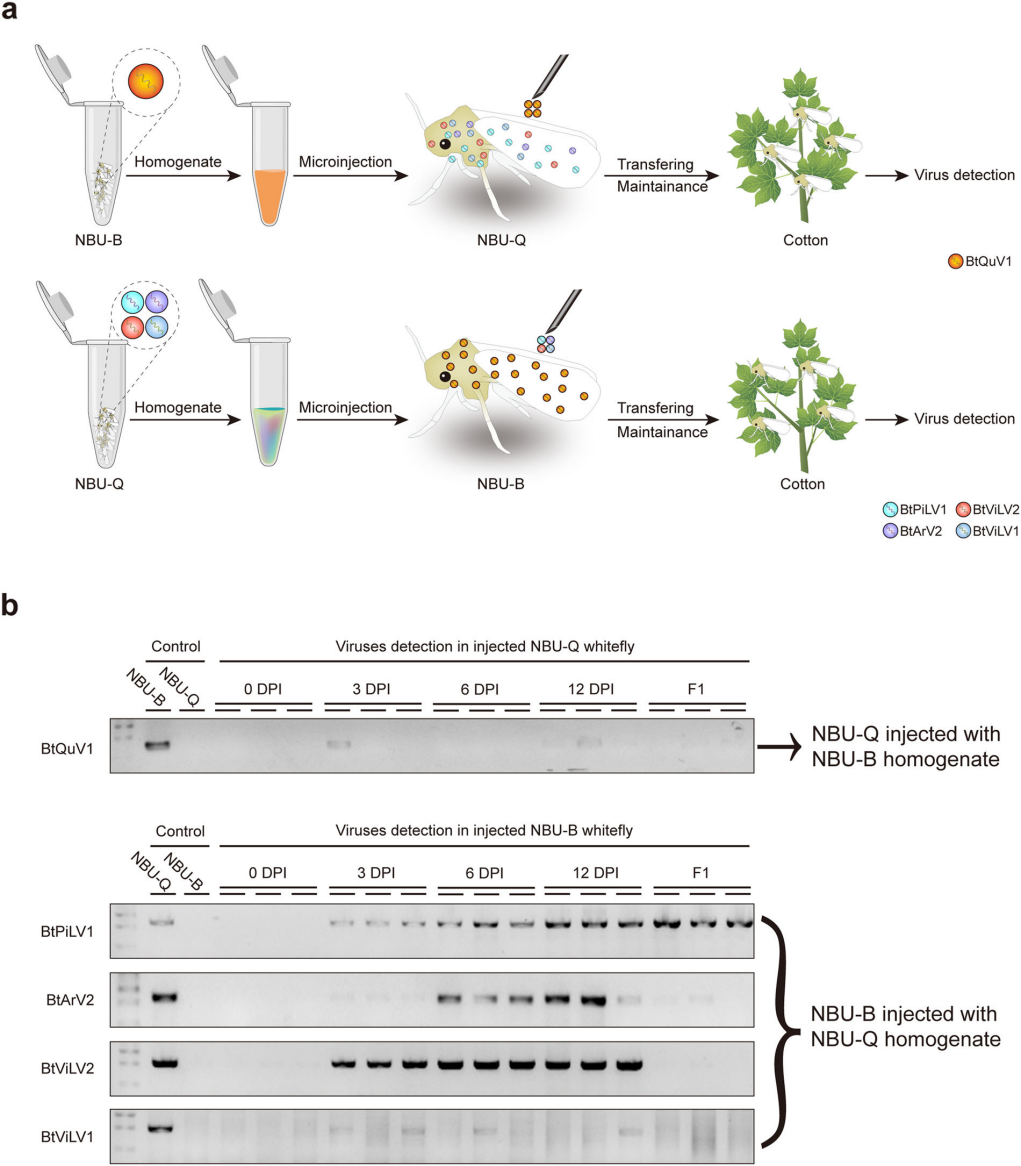
Evaluation of the ability of insect-specific viruses to cross-infect the cryptic whitefly species Middle East Asia Minor 1 (MEAM1) and Mediterranean (MED). **a** Diagram illustrating the experimental workflow. In the upper panel whitefly individuals of NBU-Q (representing cryptic species MED) were microinjected with homogenate of NBU-B (representing cryptic species MEAM1). The reciprocal injection of NBU-B with homogenate of NBU-Q whiteflies is shown in the lower panel. **b** Detection of insect-specific viruses (ISVs) 0, 3, 6, and 12 days post injection (DPI), and in the next generation (F1). The presence of ISVs in each whitefly sample was determined by RT-PCR. Untreated NBU-B and NBU-Q whiteflies were used as controls. A pool of 20–30 whiteflies were collected for MEAM1 and MED whiteflies at each time point, and three independent biological replicates were performed. Abbreviation of virus names: *BtArV2*, Bemisia tabaci arlivirus 2, *BtPiLV1* Bemisia tabaci picorna-like virus 1, *BtQuV1* Bemisia tabaci quaranjavirus 1, *BtViLV1* Bemisia tabaci virga-like virus 1, *BtViLV2* Bemisia tabaci virga-like virus 2.

Since most of whitefly ISVs were identified from only one specific cryptic species, we next tested whether the whitefly ISVs of the cryptic species MEAM1 and MED were able to cross-infect. The whitefly population NBU-B (representing of cryptic species MEAM1) and NBU-Q (representing of cryptic species MED) in our lab were used for this study through microinjection as described in “Methods” and Fig. 4a. NBU-B contains only one virus BtQuV1 that is the dominant virus in MEAM1, while the four ISVs, BtPiLV1, Bemisia tabaci arlivirus 2 (BtArV2), BtViLV2, and Bemisia tabaci virga-like virus 1 (BtViLV1) present in NBU-Q appear to be “MED-specific” (Fig. 3). Results of RT-PCR and qRT-PCR detection indicated that BtQuV1 could barely be detected in NBU-Q microinjected with whitefly homogenate (NBU-B) 0, 3, 6, and 12 DPI. In addition, nearly no viral RNA of BtQuV1 was detected in the F1 of injected NBU-Q, confirming that the core virus of MEAM1 (BtQuV1) might not successfully replicate and be transmitted transovarially in a MED whitefly population (Fig. 4b and Supplementary Fig. 7e). When the four “MED-specific” ISVs were injected into NBU-B, BtPiLV1 replicated well with increasing accumulation of detected viral RNAs, and the virus could also be easily identified in the F1 of NBU-B whiteflies, providing the possibility of its ability to replicate and of transovarial transmission in MEAM1 whiteflies (Fig. 4b and Supplementary Fig. 7a). BtArV2 and BtViLV2 were also present at 3, 6, and 12 DPI in NBU-B, providing evidence of replication in MEAM1 but they could hardly be detected in the F1 of NBU-B whiteflies, suggesting that they might not be transmitted in a transovarial manner (Fig. 4b and Supplementary Fig. 7b, c). For BtViLV1, only several weak bands were detected (likely the injected virus) in some of the NBU-B whiteflies and no virus could be detected in the F1 generation, indicating that BtViLV1 may be MED-specific and unable to replicate in MEAM1 whiteflies (Fig. 4b and Supplementary Fig. 7d).

### Analysis of virus-derived siRNAs in *B. tabaci*

**Fig. 5:**
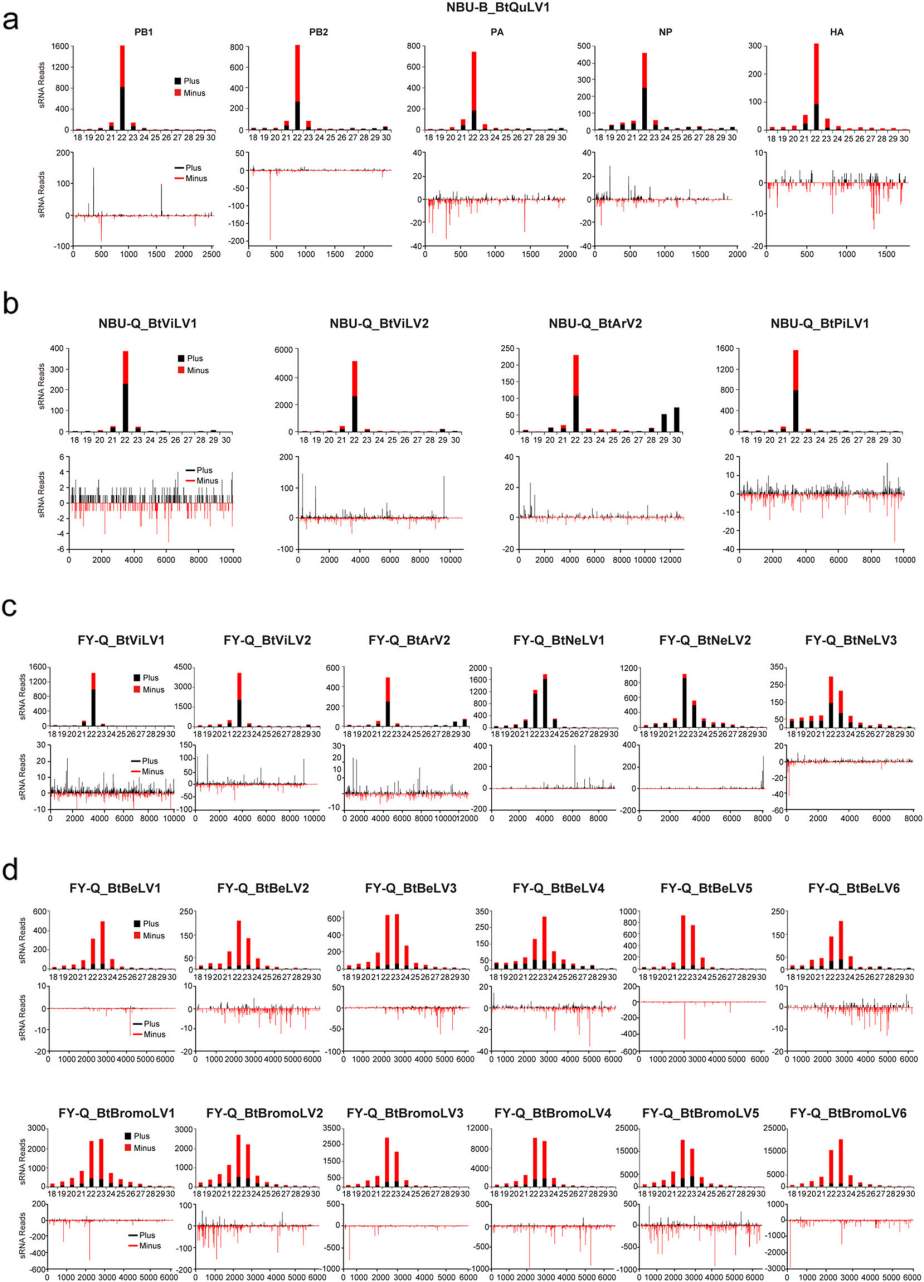
Profiles of virus-derived small interfering RNAs (vsiRNAs). vsiRNAs derived from whitefly datasets NBU-B (**a**), NBU-Q (**b**), and FY-Q (**c**, **d**). The upper panel shows the size distribution of vsiRNAs, while the lower panel shows the distribution of vsiRNAs along the corresponding viral genome. Color coding shows vsiRNAs derived from the sense (black, plus) and antisense (red, minus) genomic strands. All reads in this analysis are redundant. The abbreviation of the virus names is listed in Table 2.

To further explore siRNA-based antiviral immunity in *B. tabaci*, virus-derived siRNAs (vsiRNAs) from the whitefly datasets of NBU-B (BtQuLV1), NBU-Q (BtViLV1, BtViLV2, BtArV2, and BtPiLV1), and FY-Q (BtViLV1, BtViLV2, BtArV2, BtNeLV1-3, BtBeLV1-6, and BtBromoLV1-6) were comprehensively characterized. For BtQuLV1 in the whitefly dataset NBU-B, siRNAs derived from the five segments are mostly 22 nt long. vsiRNAs are almost equally derived from the sense and antisense strands of the viral genomic RNA in segments PB1 and NP, whereas more sense vsiRNAs are detected for PB2, PA, and HA (Fig. 5a). The other three ISVs (BtViLV1, BtViLV2, BtArV2) in both datasets of NBU-Q and FY-Q, and BtPiLV1 in NBU-Q, are also mostly 22 nt long and are equally derived from both vsiRNA strands (Fig. 5b, c). The typical size distribution and polarity of vsiRNAs strongly suggested that these ISVs can successfully replicate in whitefly, and the antiviral RNAi pathway of *B. tabaci* is actively involved in response to ISV infections. In contrast, for BtNeLV1-3, BtBeLV1-6, and BtBromoLV1-6 there are many more 23nt vsiRNAs (Fig. 5c, d) and the vast majority of siRNAs of BtNeLV1 and BtNeLV2 are from the positive strand of the genome (Fig. 5c), while there is an obvious preference of vsiRNAs derived from antisense strands of the viral genomes of BtBeLV1-6 and BtBromoLV1-6. The discrepant and non-canonical characteristics of vsiRNAs derived from BtNeLV1, BtNeLV2, BtBeLV1-6, and BtBromoLV1-6 raises the possibility that these vsiRNAs (or perhaps some of them) may be produced by the microorganism/parasitism of the insect host, rather than directly from cleavage by the whitefly siRNA immune pathway. Moreover, our analysis showed a strong U bias in the 5′-terminal nucleotide of vsiRNAs for BtNeLV1-3, BtBeLV1-6, and BtBromoLV1-6 (Supplementary Fig. 8c) in whitefly FY-Q, whereas classical A/U bias of vsiRNAs was observed for the other viruses (BtQuLV1, BtViLV1, BtViLV2, BtArV2, and BtPiLV1) discovered in the three whitefly datasets (Supplementary Fig. 8). Previous study indicated that the distinct patterns of vsiRNAs produced by various hosts can be used for virus detection^27^. An unusual profile of vsiRNAs was also reported for a Twyford virus discovered in *D. melanogaster*^28^, and more recently, a study showed that non-canonical characteristics of these vsiRNAs (21–23 peak, negative strand bias, and a strong 5′ U bias) were more likely to be processed by a Dicer pathway in a fungi rather than insect host^29^, indicating that BtBeLV1-6 and BtBromoLV1-6 might be originated from fungi of whitefly (Fig. 5 and Supplementary Fig. 8). In addition, analysis of the distribution of vsiRNAs shows them to be widely distributed in the corresponding genomes (segments) but with notable asymmetric hotspots on both strands, which may indicate that these regions are preferentially targeted for cleavage by the host immune system (Fig. 5).

## Discussion

Over the past decade, a large number of insect-associated viruses (mostly ISVs) have been discovered by taking advantage of mNGS and advanced bioinformatics tools, contributing new insights on insect viromes and viral evolution^5^. In this study, the RNA virome of a notorious insect pest, the whitefly *B. tabaci*, and the complex association of ISVs with its cryptic species were investigated by screening the publicly available whitefly datasets, as well as the whitefly populations derived from lab and field populations in China. Twenty novel ISVs, together with an isolate of a plant RNA virus and 12 novel plant/fungal virus-like contigs were identified among these whitefly samples. All the ISVs discovered in whitefly are novel and distinct from previously described ones (Table 2), suggesting the rich diversity of ISVs in whiteflies and that different ISVs may be associated with specific hosts. In addition, our results add a number of new virus members to unclassified groups which will facilitate the official establishment of new viral taxa in the future. These include unclassified clades in *Flaviviridae* (Fig. 2c), Martellivirales (Fig. 2d), Picornavirales (Fig. 2e), and *Totiviridae* (Fig. 2f). Previous studies have shown that it is common for a host insect to harbor several closely related ISVs, such as partitiviruses in *Drosophila*^28^, negeviruses in a dungfly^30^, and totiviruses, anpheviruses, and quaranja-like viruses in mosquitoes^17^. Our results also discovered that a number of intimately related viruses were present in different datasets of whitefly, including two arliviruses (Figs. 1a and 2a), three nege-like viruses (Figs. 1d and 2d), two picorna-like viruses (Figs. 1g and 2e), and three quaranjaviruses (Figs. 1i and 2g). The three quaranjaviruses discovered in whitefly are of particular interest because our results indicate that the diversity of quaranjaviruses in the family *Orthomyxoviridae* might be greatly underestimated in arthropods other than the previously described hematophagous insects.

The majority of economically important plant viruses are transmitted by insect vectors, and the recently developed “vector-enabled metagenomics” method facilitates the discovery of new plant viruses or new insect vectors of known plant viruses^31,32^. Our detection of a new isolate of PVS in the dataset CU-B1 was unexpected. Several different species of aphids, including *Myzus persicae* and *Aphis nasturtii*, are well-known to be efficient vectors for the transmission of PVS^33,34^ and our results provide the evidence that whiteflies might also be vectors of PVS, although further field investigations and lab experiments are needed to confirm it. In view of the relatively low coverage (20×) of PVS, it is also possible for the accident acquisition of PVS by the whitely. Nevertheless, understanding the complex diversity of plant viruses in insect vectors will contribute to the early surveillance of emerging plant viruses and the management of viral diseases.

Previous studies have shown that the viromes of the two important mosquitoes, *A. aegypti* and *C. quinquefasciatus*, have their own relatively stable core virome, which might have important implications for the competence to host the relevant arboviruses^17^. Moreover, further investigation revealed that the core virome was very stable across all developmental stages of both lab-derived and field-collected *A. albopictus*^35^. Our results suggested that different cryptic species of *B. tabaci* clearly harbor specific core virus/viruses (Fig. 3b), indicating a long-term coevolution between these ISVs and cryptic species of whitefly. Because the majority of the whitefly datasets used in this study are from public databases and are mostly derived from lab cultures, it is expected that the cryptic-species-specific core viruses in these whitefly cultures might constitute a vertically transmitted core virome, whereas the field whitefly dataset (FY-Q) likely harbors distinct environmentally-derived viruses (BtNeLV1-3, BtBeLV1-6, and BtBromoLV1-6) as described previously^35^. Nevertheless, more investigation is needed to confirm this hypothesis due to limited whitefly datasets from the field used in this study. It should also be noted that the dominant ISVs of MED whitefly exhibit more diversity and are associated directly with the original location of the whitefly as well as its taxonomical status (Fig. 3b), implying that other dominant/core viruses might be obtained from the environment and establish stable infections in the local whitefly populations.

Cross-species transmission (or interspecies transmission) is the ability for a foreign virus to infect a new host species individually and spread in the new host population^36^. Interspecies transmission has been well demonstrated in several important emerging zoonotic viruses, including Severe Acute Respiratory Syndrome (SARS-CoV), Ebola, swine flu, rabies, avian influenza, as well as SARS-CoV-2, the causative agent of the current COVID-19 outbreak^37,38^. Our study showed that most of the ISVs discovered in this study exhibit close association with a specific cryptic species, but some have the ability to infect different cryptic species (Fig. 3c). Microinjection experiments confirmed that the “MEAM1-specific” virus BtQuV1 cannot establish infection or be transmitted in a transovarial manner in MED whitefly, whereas some “MED-specific” ISVs (BtPiLV1, BtArV2, BtViLV2, and BtViLV1) could replicate and sometimes be transmitted transovarially in MEAM1 whitefly (Fig. 4). The distinct interspecies transmission abilities of ISVs in MED and MEAM1 whitefly might reflect the different origin and discrete long-term coevolution of these ISVs with their whitefly hosts. Further investigations are necessary to answer the intriguing issue about how some, but not all, of these ISVs can cross the cryptic species barrier of whitefly. In addition, the high diversity and efficient cross-species ability of some ISVs in various cryptic whitefly species may provide an excellent model system for future studies on the molecular and evolutionary mechanisms of interspecies transmission in zoonotic viruses. However, more investigations are needed to confirm the complex relationships between the ISVs and whitefly cryptic species, and to comprehensively evaluate and understand the ability of ISVs to cross the cryptic species boundaries.

One of the most important reasons that ISVs have gained increasing attention recently is because they can affect vector competence and could therefore have potential as biocontrol agents. Previous studies in mosquitos indicated that ISVs can negatively regulate several arbovirus infections both in vitro and in vivo^5,39^. For example, two ISVs identified in an *A. albopictus* C6/36 cell line, Menghai rhabdovirus and Shinobi tetravirus, suppressed Zika virus replication in vitro^40^. It is proposed that the infection of ISVs might indirectly upregulate the innate immune system of mosquitoes and further interfere with the replication of mosquito-borne pathogenic viruses by decreasing vector competence^5,39,41,42^, which is similar to the molecular mechanism by which *Wolbachia* controls arboviruses in mosquitos^43^. The activation of the siRNA-based antiviral response by ISVs in whiteflies (Fig. 5) indicates that the immune system is also induced and raises the possibility that this might interfere with the transmission of devastating plant viruses, including begomoviruses, which are vectored by whiteflies. Our results also highlight the need to recognize that ISVs and other viruses in an insect vector could have important effects on laboratory experiments, especially for studies related to the immune response of vector insects challenged by various pathogens. It would be important, therefore, to investigate and understand the virome background of any insect line maintained in the lab, particularly where it is being used in vector studies.

## Methods

### RNA sequencing (RNA-seq) libraries of *B. tabaci* from the public database

Information about approximately 400 RNA-seq datasets of *B. tabaci* was retrieved from the NCBI SRA repository. Filtering of the datasets was based on the following criteria: Firstly, the dataset should be >4 Gb since RNA-seq depth is essential for virus discovery; secondly, where there were several biological replicates, the dataset with largest total number of bases was selected (since a similar virome should be present within the replicates); thirdly, the datasets were not used unless some novel virus was identified. Exceptions to these rules included a dataset of 3.4 Gb (PRJDB2008), representing a unique submitter (National Institute of Agrobiological Sciences, Tsukuba, Japan) and 12 datasets with the project number PRJNA427517 (each with total bases <3.0 Gb) submitted by The Hebrew University, Jerusalem, Israel, because the project included several different cryptic species of *B. tabaci* that were of interest for this study. In total, 41 high-quality SRA datasets representing the different whitefly cryptic species and various geographical locations worldwide were chosen for further bioinformatics analysis. Abbreviations and detail information of these whitefly datasets are provided in Table 1.

### RNA-seq libraries of *B. tabaci* generated from lab and field samples

The *B. tabaci* culture of cryptic species MEAM1 (NBU-B) was kindly provided by Xiao-Wei Wang and Shu-Sheng Liu (Institute of Insect Sciences, Zhejiang University) in June 2019, and the MED population of *B. tabaci* (NBU-Q) maintained in our lab at Ningbo University (NBU) was originally collected from soybean plants in Suzhou (An’hui province, China) in June 2019. The NBU-B and NBU-Q were maintained in the laboratory in Ningbo University thereafter. The two whitefly cultures were reared separately in insect-proof cages on cotton plants (*Gossypium hirsutum* L. cv. Zhemian 1793) at 25 ± 1 °C, 50–70% relative humidity, and 14 h light/10 h darkness. The field sample of *B. tabaci* was collected from cucumber plants in Fuyang (An’hui province, China) on November 2019 and the cryptic species was determined to be MED (FY-Q) by mtCOI sequences^23^. Total RNAs were extracted using approximately 100 adult whiteflies from each of the two lab cultures (NBU-B, NBU-Q), as well as the field sample (FY-Q). Each RNA sample was subdivided for Illumina high throughput sequencing (transcriptome and sRNA). Specifically, for transcriptome, paired-end (150 bp) sequencing of the RNA library was performed on the Illumina HiSeq 4000 platform (Illumina, CA, USA) by Novogene (Tianjin, China). For sRNA, the cDNA libraries were prepared using the Illumina TruSeq Small RNA Sample Preparation Kit (Illumina, CA, USA), and sRNA sequencing was performed on an Illumina HiSeq 2500 by Novogene (Tianjin, China). The transcriptome raw reads of NBU-B, NBU-Q, and FY-Q were deposited in SRA under accession numbers SRR13050950, SRR13052369, and SRR13039280, respectively. Meanwhile, the sRNA raw reads of NBU-B, NBU-Q, and FY-Q were deposited in SRA under accession numbers SRR13050947, SRR13050948, and SRR13082984, respectively.

### Dataset reassembly and assignment of whitefly cryptic species

Raw reads of the 41 selected datasets from the SRA repository, as well as the transcriptomes of the lab populations (NBU-B, NBU-Q) and field sample (FY-Q), were quality trimmed. The remaining reads were reassembled/assembled de novo using the two assembler software packages Trinity and MetaviralSPAdes with default parameters^44,45^. The assembled contigs were searched against the mtCOI reference database of the *B. tabaci* species complex to assign the correct cryptic species of whitefly for each dataset^46^. Furthermore, to facilitate the discovery of the whitefly RNA virome, the host-derived reads were removed by mapping against the two representative genomes of whitefly (GCA_001854935.1 and GCA_003994315.1) using BWA software^47^.

### RNA virome discovery

All the assembled contigs were compared with the NCBI viral RefSeq database using diamond Blastx^48^. Since most of the datasets were retrieved from public databases, strict criteria were used for the identification of putative novel viruses in each dataset. Firstly, *E*-value cutoff of 1 × 10^−20^ was rigorously set for the diamond Blastx. Secondly, the minimum coverage and length threshold for the viral homology contigs was no less than 20× and 500 bp, respectively, and the viral homology contigs had to contain almost complete open reading frames (ORF) of predicted viral proteins. Thirdly, the viral homology contigs needed to be confirmed by both of the assemblers (Trinity and MetaviralSPAdes). Finally, the regions of the candidate viral-like contigs matched to the reference virus were extracted and further compared with the entire NCBI nucleotide and non-redundant protein databases to eliminate false positives. In addition, RT-PCR followed by Sanger sequencing was performed to verify the presence of the newly discovered viruses in the whitefly populations NBU-B, NBU-Q, and FY-Q. The primers used for RT-PCR are listed in Supplementary Table 1. Sequences of all identified novel viruses from this study have been deposited in GenBank (MW256664–MW256706 and MW227222–MW227223).

### Viral genome annotation and phylogenetic analysis

The newly identified viral contigs were annotated with InterPro^49^. Conserved RNA-dependent RNA polymerase (RdRp) regions of the discovered viruses, together with RdRp protein sequences of reference viruses, were used for phylogenetic analysis. The RdRp sequences were aligned with MAFFT^50^, and ambiguously aligned regions were trimmed by Gblock^51^. The best-fit model of amino acid substitution was evaluated by ModelTest-NG^52^. Maximum likelihood (ML) trees were constructed using RAxML-NG with 1000 bootstrap replications^53^. Details of all the reference sequences used in phylogenetic analysis are listed in Supplementary Table 2.

### Correlation between whitefly cryptic species and ISVs

Phylogeny of whitefly cryptic species was constructed as described above. For the identification of ISV-derived viral reads, raw reads of each whitefly dataset were aligned to corresponding ISV contigs using bowtie2 software^54^. To better understand the relative abundance of the newly identified ISVs across the different whitefly datasets, the unassembled transcriptome reads of each datasets were mapped back to the corresponding viral contigs. Specifically, relative abundance for the ISV of each whitefly dataset was calculated and normalized based on the transcripts per million (TPM) values calculated as $a_j = \frac{{b_j/c_j}}{{\mathop {\sum }\nolimits_{j = 1}^n b_j/c_j}}\times10^6$. In this equation, $a_j$ represents the TPM of viral contig *j*, $b_j$ represents the number of uniquely mapped fragments in a dataset, $c_j$ represents the length of viral contig *j*, and *n* is the total number of viral contigs^55,56^. In addition, relative abundance of the ISV in each whitefly dataset was further subjected to PCA using R 3.5.

### Ability of ISVs to cross-infect the whitefly cryptic species MEAM1 and MED

The whitefly lab cultures of NBU-B (MEAM1) and NBU-Q (MED) were used to investigate whether the ISVs were specific to one cryptic species of whitefly or able to infect both. Before the experiment, the presence of viruses in each cryptic species was verified by RT-PCR. To explore the potential ability of ISVs in NBU-B to infect NBU-Q whiteflies, a pool of 10 NBU-B whiteflies were homogenized in 150 μl phosphate-buffered saline solutions (137 mM NaCl, 2.68 mM KCl, 8.1 mM Na_2_HPO_4_, and 1.47 mM KH_2_PO_4_ at pH 7.4). After centrifugation at 12,000 r.p.m. for three times, the supernatant was collected and microinjected into individual NBU-Q whiteflies (approximately 0.02 μl/per insect) as described previously^57^. The injected whiteflies were then transferred and maintained on cotton plants. The same method was also used in a reciprocal manner to evaluate the potential ability of ISVs in NBU-Q to infect NBU-B. The microinjected whiteflies were collected at 0, 3, 6, and 12 days post injection (DPI) and the viruses were detected by RT-PCR and qRT-PCR. Additionally, the microinjected whiteflies were allowed to oviposit, and the presence of ISVs in the next generation (F1) were also determined using RT-PCR and qRT-PCR. For qRT-PCR analysis, primers were designed using Primer Premier v6.0, and the *B. tabaci* 18sRNA was used as an internal control. The reaction was run on a Roche Light Cycler^®^ 480 Real-Time PCR System using the SYBR Green Supermix Kit (Yeasen, Shanghai, China) under the following programs: denaturation for 5 min at 95 °C, followed by 40 cycles at 95 °C for 10 s and 60 °C for 30 s. A relative quantitative method (2^−ΔΔCt^) was used to evaluate quantitative variation. A pool of 20–30 whiteflies were collected for MEAM1 and MED at each time point, and three independent biological replicates were performed. The primers for RT-PCR and qRT-PCR are listed in Supplementary Table 1.

### Tissue expression of ISVs in whitefly NBU-Q and NBU-B

To investigate the relative spatial expression of ISVs in *B. tabaci*, tissue samples from salivary glands, guts, fat bodies, ovaries, and carcasses were dissected from the whitefly lab cultures of NBU-Q and NBU-B in a phosphate-buffered saline (PBS) solution (137 mM NaCl, 2.68 mM KCl, 8.1 mM Na_2_HPO_4_, and 1.47 mM KH_2_PO_4_ at pH 7.4) under a stereomicroscope (Olympus SZX7, Tokyo, Japan) using sharp forceps (Ideal-Tek, Switzerland). The collected samples were immediately transferred to TRIzol Reagent (Invitrogen Corp., CA, USA) using Eppendorf tips. After RNA extraction, the relative abundance of ISVs in each tissue was determined by qRT-PCR as described above.

### Small RNA analysis

The sRNA raw reads of the three libraries NBU-B, NBU-Q, and FY-Q were first treated to remove the adapter, low quality, and junk sequences as described previously^58^. The clean sRNA reads 18- to 30-nt long were extracted using the FASTX-Toolkit (http://hannonlab.cshl.edu/fastx_toolkit). The extracted sRNA were then mapped to the identified viral contigs using Bowtie software with perfect match (i.e. allowing zero mismatch)^59^. Downstream analyses were performed using custom perl scripts and Linux shell bash scripts.

### Reporting summary

Further information on research design is available in the Nature Research Reporting Summary linked to this article.

## Supplementary information

Supplementary Information

Reporting Summary

## Data Availability

The raw reads of RNA-seq generated in this study were deposited in NCBI SRA with accession numbers SRR13050950 (NBU-B), SRR13052369 (NBU-Q), SRR13039280 (FY-Q) for transcriptome, and SRR13050947 (NBU-B), SRR13050948 (NBU-Q), SRR13082984 (FY-Q) for sRNA, respectively. Sequences of all identified novel viruses from this study have been deposited in NCBI GenBank with accession numbers MW256664–MW256706 and MW227222–MW227223.
