# Supplementary material for: Diversity and infectivity of the RNA virome among different cryptic species of an agriculturally important insect vector: whitefly Bemisia tabaci
Source: NPJ Biofilms Microbiomes. 2021 May 13;7:43. doi: 10.1038/s41522-021-00216-5 (PMC8119434; doi:10.1038/s41522-021-00216-5)
Supplement: Supplementary file 1 — Supplementary Information [file 41522_2021_216_MOESM1_ESM.pdf]

Supplementary Information for:

Diversity and infectivity of the RNA virome among different cryptic species of an agricultural important insect vector: whitefly *Bemisia tabaci*

Hai-Jian Huang[#], Zhuang-Xin Ye[#], Xin Wang, Xiao-Tian Yan, Yan Zhang, Yu-Juan He, Yu-Hua Qi, Xiao-Di Zhang, Ji-Chong Zhuo, Gang Lu, Jia-Bao Lu, Qian-Zhuo Mao, Zong-Tao Sun, Fei Yan, Jian-Ping Chen[*], Chuan-Xi Zhang[*] and Jun-Min Li[*]

State Key Laboratory for Managing Biotic and Chemical Threats to the Quality and Safety of Agro-products, Key Laboratory of Biotechnology in Plant Protection of Ministry of Agriculture and Zhejiang Province, Institute of Plant Virology, Ningbo University, Ningbo 315211, China

[*]Authors for correspondence:

Jian-Ping Chen, jpchen2001@126.com;

Chuan-Xi Zhang, chxzhang@zju.edu.cn;

Jun-Min Li, lijunmin@nbu.edu.cn;

[#]These authors contribute equally to this work.

Contents:

Supplementary Figure 1. Global distribution of SRA submitters of whitefly datasets used in this study.

Supplementary Figure 2. Average nucleotide and amino acid identity among three *Bemisia tabaci* quaranja-like viruses.

Supplementary Figure 3. Genomic structures and phylogeny of the other novel viral contigs discovered in whitefly *Bemisia tabaci*.

Supplementary Figure 4. Average nucleotide identity for viral-like contigs in whitefly *Bemisia tabaci*.

Supplementary Figure 5. Relative expression of insect-specific viruses in different tissues of whitefly datasets NBU-Q and NBU-B.

Supplementary Figure 6. Principal component analysis (PCA) of the relative abundance of RNA viruses in whitefly cryptic species.

Supplementary Figure 7. Quantitative reverse transcription PCR (qRT-PCR) analysis of the insect-specific viruses in the whitefly datasets NBU-Q and NBU-B using the same RNA samples in Fig. 4b.

Supplementary Figure 8. Distribution of the 5′-terminal nucleotide of virus-derived small interfering RNAs (vsiRNAs) in the whitefly datasets NBU-B (a), NBU-Q (b), and FY-Q (c).

Supplementary Result 1. Descriptions of novel viruses identified in whitefly, *Bemisia tabaci*.

Supplementary Table 1. Primers used in this study.

Supplementary Table 2. Details of reference viruses extracted from GenBank for phylogenetic analysis.

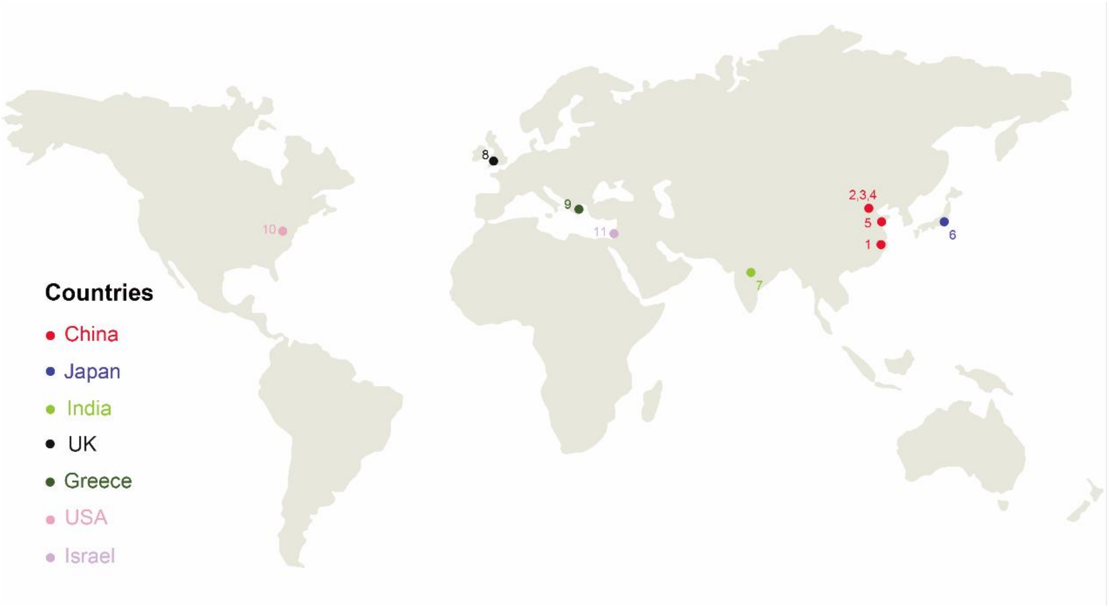

Supplementary Figure 1

Global distribution of SRA submitters of whitefly datasets used in this study. 1 Zhejiang University, Hangzhou, China; 2 Chinese Academy of Agricultural Sciences, Beijing, China; 3 China Agricultural University, Beijing, China; 4 Chinese Academy of Sciences, Beijing, China; 5 Qingdao Agricultural University, Qingdao, China; 6 National Institute of Agrobiological Sciences, Tsukuba, Japan; 7 National Agri-Food Biotechnology Institute, Ajitgarh, India; 8 University of Exeter, Penryn, UK; 9 University of Crete, Heraklion, Greece; 10 Cornell University, New York, USA; 11 The Hebrew University, Jerusalem, Israel.

Nucleotide

PB2

	BtQuV1	BtQuV2	BtQuV3
BtQuV1	100	89.5	71.2
BtQuV2		100	70.7
BtQuV3			100

PA

	BtQuV1	BtQuV2	BtQuV3
BtQuV1	100	89.1	72.4
BtQuV2		100	73.2
BtQuV3			100

PB1

	BtQuV1	BtQuV2	BtQuV3
BtQuV1	100	88.8	77.4
BtQuV2		100	77
BtQuV3			100

NP

	BtQuV1	BtQuV2	BtQuV3
BtQuV1	100	87.4	68.6
BtQuV2		100	67.3
BtQuV3			100

HA

	BtQuV1	BtQuV2	BtQuV3
BtQuV1	100	90	72.3
BtQuV2		100	72.1
BtQuV3			100

Amino acid

PB2

	BtQuV1	BtQuV2	BtQuV3
BtQuV1	100	97.9	76.1
BtQuV2		100	76.8
BtQuV3			100

PA

	BtQuV1	BtQuV2	BtQuV3
BtQuV1	100	96.4	76.6
BtQuV2		100	76.8
BtQuV3			100

PB1

	BtQuV1	BtQuV2	BtQuV3
BtQuV1	100	98.6	88.2
BtQuV2		100	87.7
BtQuV3			100

NP

	BtQuV1	BtQuV2	BtQuV3
BtQuV1	100	95.6	77.3
BtQuV2		100	78.1
BtQuV3			100

HA

	BtQuV1	BtQuV2	BtQuV3
BtQuV1	100	96.6	81
BtQuV2		100	81
BtQuV3			100

Supplementary Figure 2

Average nucleotide and amino acid identity among three Bemisia tabaci quaranja-like viruses. The pairwise distances between nucleotide sequences (left) and amino acid sequences (right) among five segments of Bemisia tabaci quaranja-like virus 1-3 (BtQuV1-3) calculated using MegAlign from the DNASTAR Lasergene 12 software package.

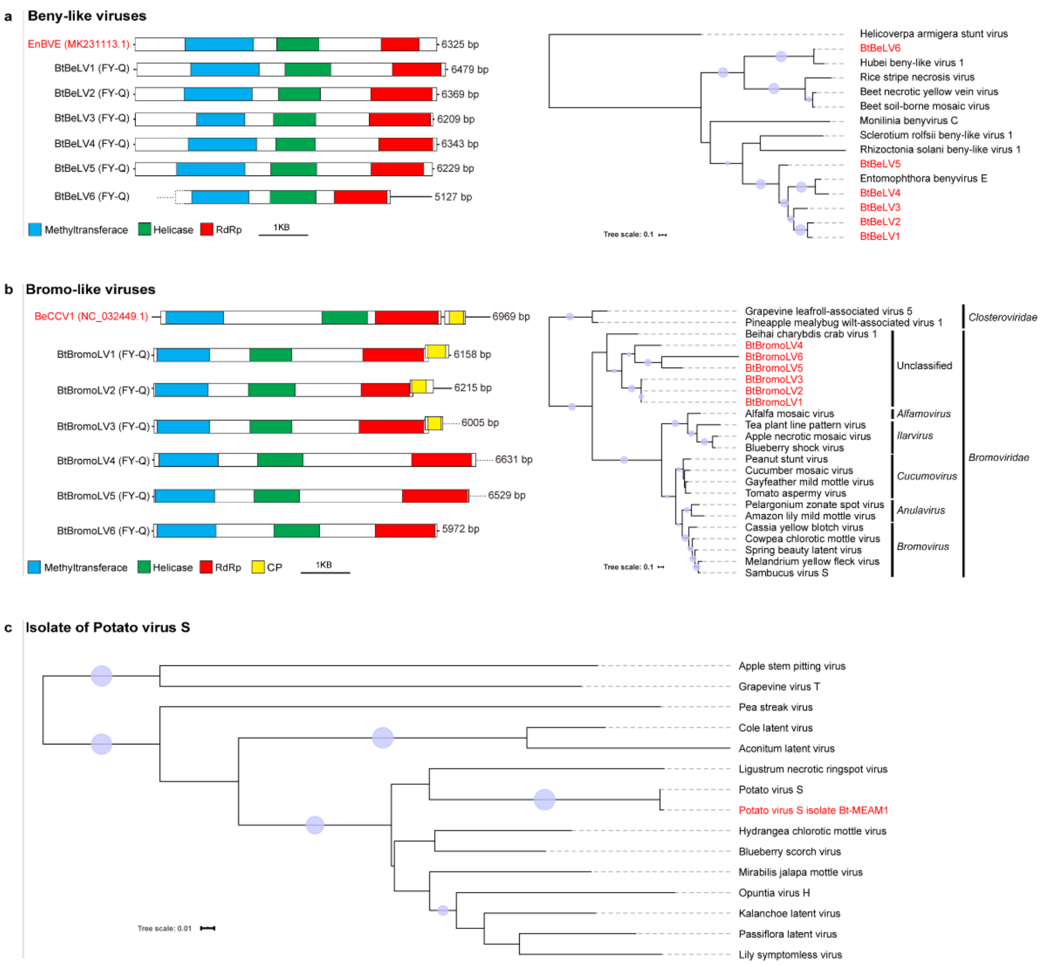

Supplementary Figure 3

Genomic structures and phylogeny of the other novel viral contigs discovered in whitefly *B. tabaci*. These viruses were identified as Beny-like viruses (a), Bromo-like viruses (b), and an isolate of Potato virus S (c). Panels A and B show a genome of a closely-related reference virus at the top (with red fonts) and the viral contigs discovered in this study below. Conserved functional domains are color-coded and the names of the domains are indicated at the bottom of the panel. Abbreviations of the virus names: BeCCV1, Beihai charybdis crab virus 1; BtBeLV1-6, Bemisia tabaci beny-like virus 1-6; BtBromLV1-6, Bemisia tabaci bromo-like virus 1-6; EnBVE, Entomophthora benyvirus E. Abbreviation of the conserved domain names: CP, Coat protein; RdRp, RNA-dependent RNA polymerase. Phylogenic trees were constructed based on the maximum likelihood method and inferred from viral RdRp domains. The novel viral contigs identified in this study are shown in red font. Nodes with bootstrap values > 50% are marked with solid blue circles, and the larger circles indicate higher bootstrap values. The viral sequences used in this study were extracted from GenBank. Accession numbers and other related details are listed in Supplementary Table 2.

a

	BtBeLV1	BtBeLV2	BtBeLV3	BtBeLV4	BtBeLV5	BtBeLV6
BtBeLV1	100	73.7	60.8	51.8	57.8	38.6
BtBeLV2		100	61.6	52.3	57.2	39.2
BtBeLV3			100	53	58.6	38.6
BtBeLV4				100	51.2	37.4
BtBeLV5					100	38.9
BtBeLV6						100

b

	BtBromLV1	BtBromLV2	BtBromLV3	BtBromLV4	BtBromLV5	BtBromLV6
BtBromLV1	100	87.5	74.4	39.4	44.9	38.7
BtBromLV2		100	74.1	39.1	45	38.2
BtBromLV3			100	39.4	44.6	38.3
BtBromLV4				100	41	38.3
BtBromLV5					100	36.8
BtBromLV6						100

Supplementary Figure 4

Average nucleotide identity for viral-like contigs in whitefly *Bemisia tabaci*. The pairwise distances between nucleotide sequences among Bemisia tabaci beny-like viruses 1-6 (BtBeLV1-6) (a) and Bemisia tabaci bromo-like viruses 1-6 (BtBromLV1-6) (b) calculated using MegAlign from the DNASTAR Lasergene 12 software package.

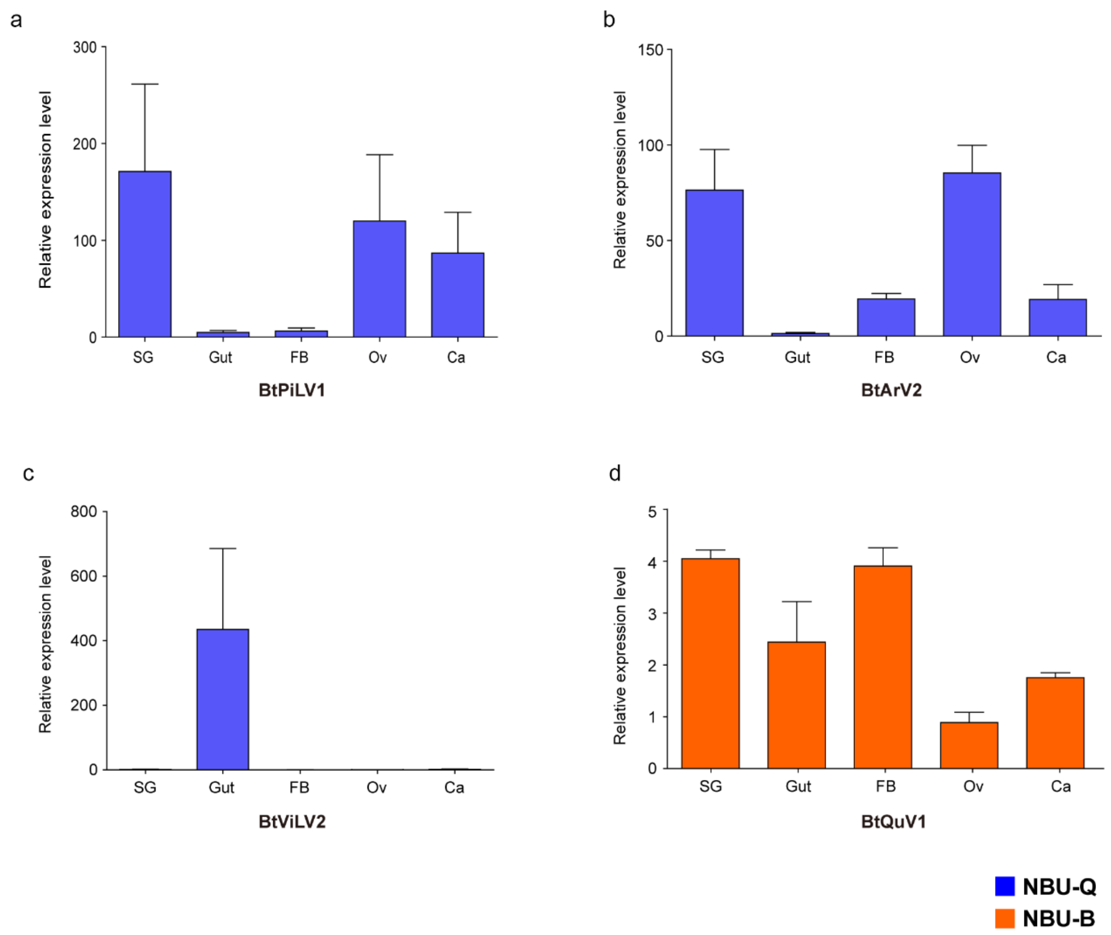

Supplementary Figure 5

Relative expression of insect-specific viruses in different tissues of whitefly datasets NBU-Q and NBU-B. Abbreviation of virus names, BtPiLV1, Bemisia tabaci picorna-like virus 1; BtArV2, Bemisia tabaci arlivirus 2; BtViLV2, Bemisia tabaci virga-like virus 2; BtQuV1, Bemisia tabaci quaranjavirus 1. Abbreviation of tissue names, SG, Salivary glands; FB, Fat bodies; Ov, Ovaries; Ca, Carcasses.

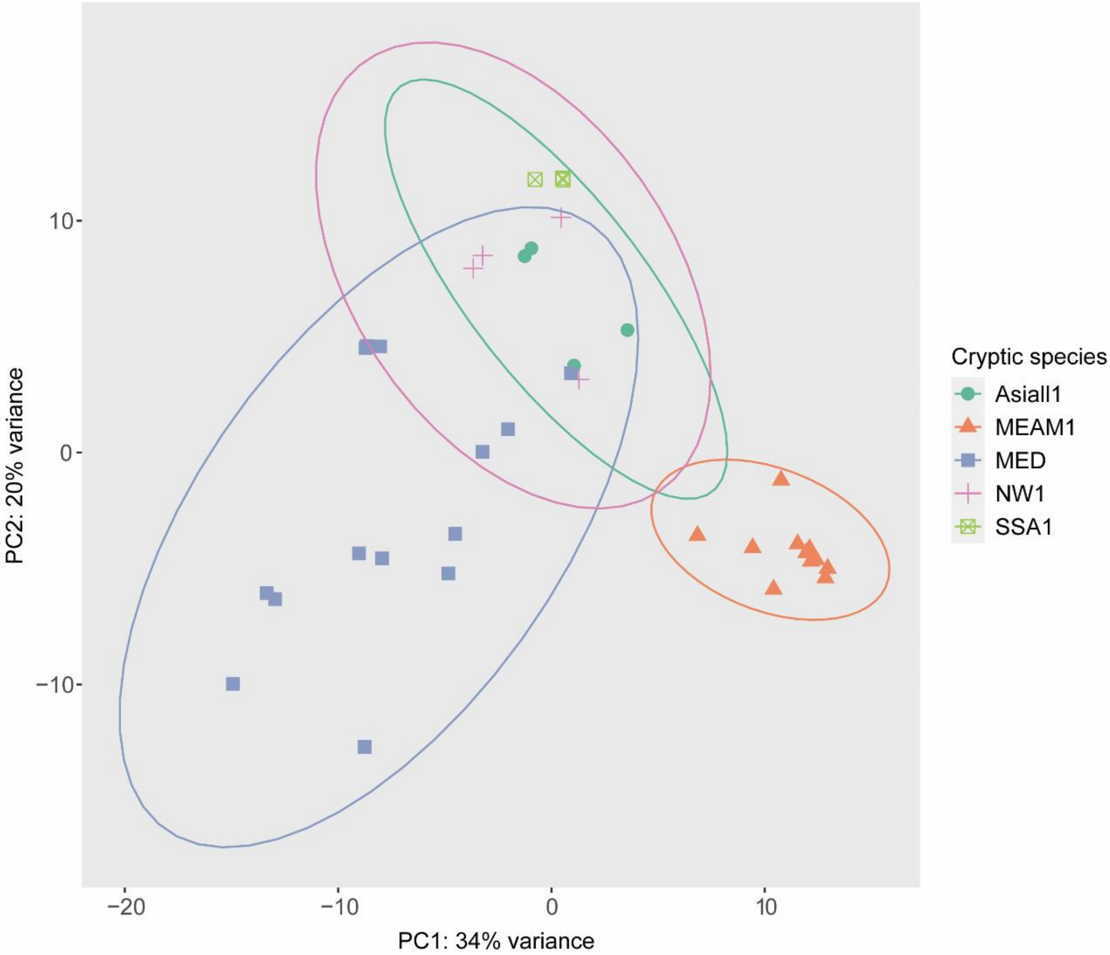

Supplementary Figure 6

Principal component analysis (PCA) of the relative abundance of RNA viruses in whitefly cryptic species. The first two principal components (PC1 and PC2) based on transcripts per million (TPM) values are shown with each plot representing the corresponding whitefly dataset. Abbreviations of the whitefly cryptic species: MEAM1, Middle East-Asia Minor 1; MED, Mediterranean; NW1, New World 1; SSA1, sub-Saharan Africa 1.

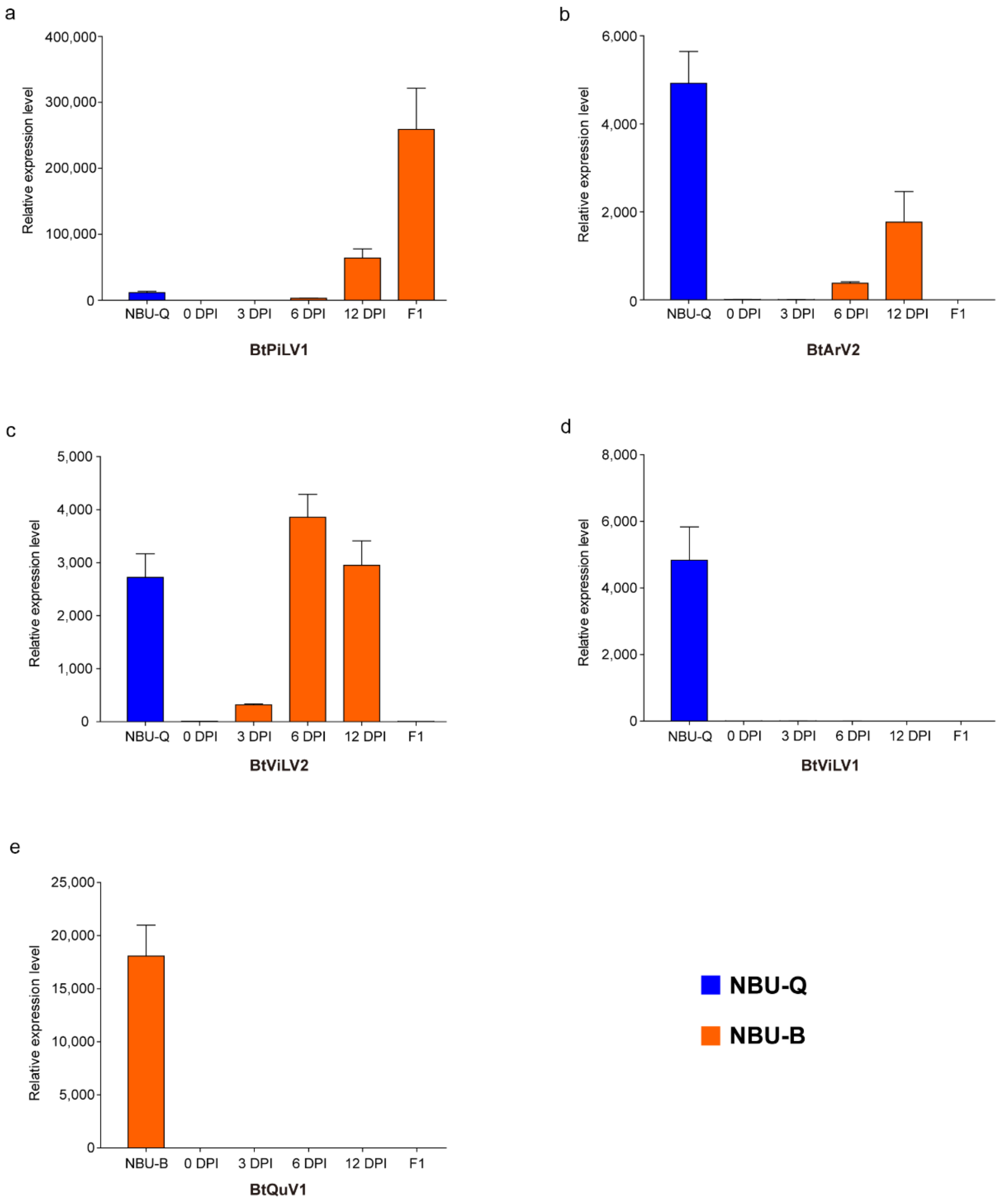

Supplementary Figure 7

Quantitative reverse transcription PCR (qRT-PCR) analysis of the insect-specific viruses in the whitefly datasets NBU-Q and NBU-B using the same RNA samples in Fig. 4b. Abbreviation of virus names, BtPiLV1, Bemisia tabaci picorna-like virus 1; BtArV2, Bemisia tabaci arlivirus 2; BtViLV2, Bemisia tabaci virga-like virus 2; BtViLV1, Bemisia tabaci virga-like virus 1; BtQuV1, Bemisia tabaci quaranjavirus 1.

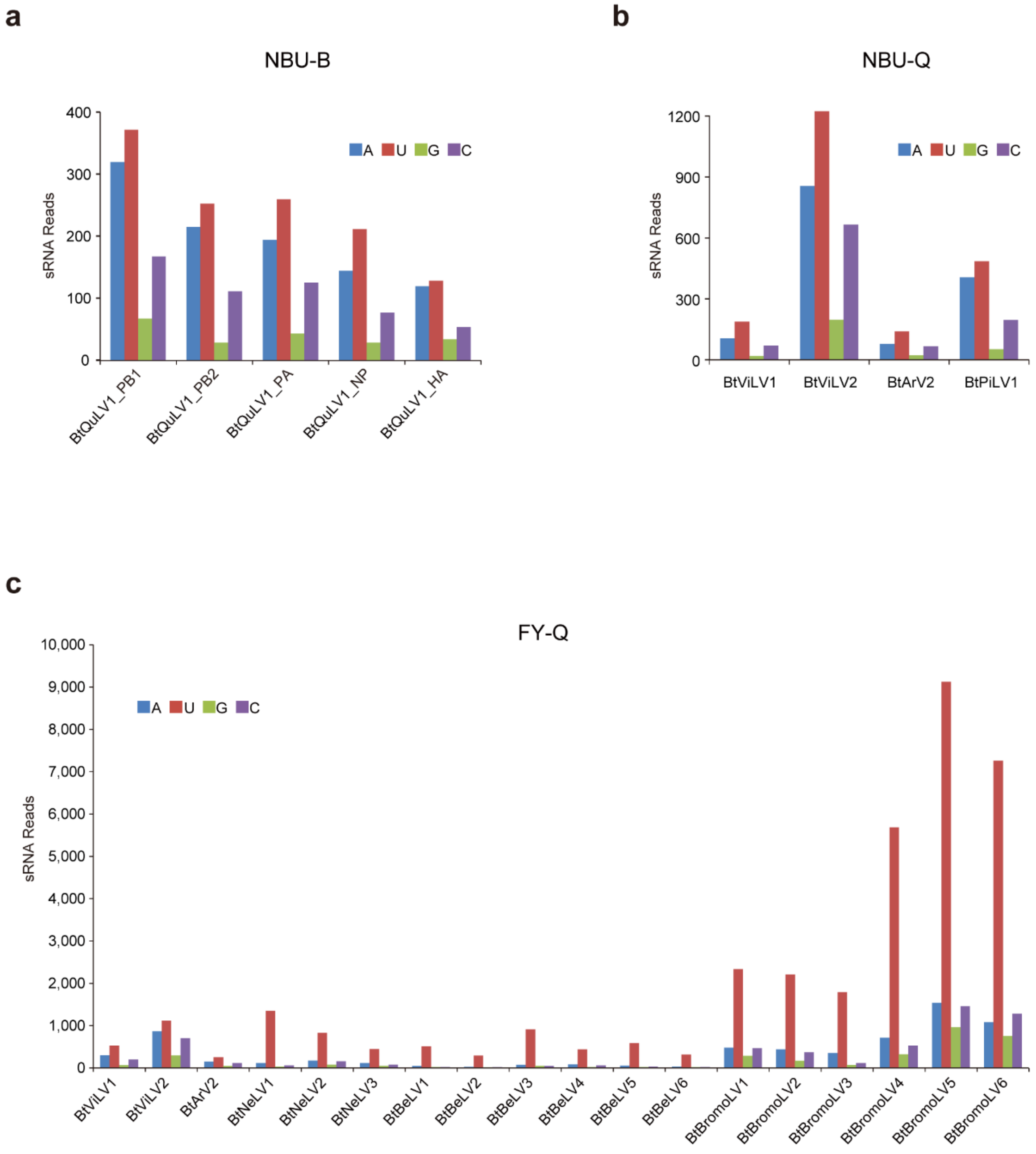

Supplementary Figure 8

Distribution of the 5′-terminal nucleotide of virus-derived small interfering RNAs (vsiRNAs) in the whitefly datasets NBU-B (a), NBU-Q (b), and FY-Q (c). All reads in this analysis are redundant. The Abbreviation of the virus names are listed in Table 2.

Supplementary Result 1

Descriptions of novel viruses identified in whitefly, *Bemisia Tabaci*

Negative-sense single-stranded RNA viruses

Two novel arliviruses with negative-sense single-stranded RNA (ssRNA) viral genomes were identified with high coverage in the libraries of ZJU-Q2 and CAU-Q1, tentatively named Bemisia tabaci arlivirus virus 1 (BtArV1, 965X) and Bemisia tabaci arlivirus virus 2 (BtArV2, 250X), respectively (Table 2). The previous floating genus *Arlivirus* is now included in the newly established family *Lispiviridae* (order Mononegavirales) and currently, six arliviruses are officially recognized by the International Committee on Taxonomy of Viruses (ICTV)[1]. The lengths of BtArV1 and BtArV2 are respectively 13,972 and 13,118 nt, representing almost the complete genomes (estimated average genome size of arliviruses is 12kb). Six open reading frames (ORFs) were predicted for both the viruses, containing one and three conserved domains identified in ORF4 and ORF6, similar to the reference arlivirus Sanxia water strider virus 4 (SxWSV4) (Fig. 1a). The ORF6 of both BtArV1 and BtArV2 encodes a replication-associated protein that contains a conserved RdRp domain, exhibiting closest homology to the RdRP of Lishi Spider Virus 2 (Accession: YP_009303699.1)[2]. In the ORF6, BtArV1 and BtArV2 are related but rather distantly (98% coverage and 36.3% amino acid identity). Phylogenetic analysis showed that BtArV1 and BtArV2 clearly cluster together with other viruses in the genus *Arlivirus*, which mainly infect invertebrates such as insects, spiders, and nematodes (Fig. 2a).

Positive-sense single-stranded RNA viruses

A novel nido-like positive-sense ssRNA virus was detected in the library CAAS-BQ with high abundance (coverage of 414X), and we provisionally named it Bemisia tabaci nido-like virus 1 (BtNiLV1). BtNiLV1 has the longest assembled contig (16,995 nt) of any of our putative new viruses. Four ORFs were found in the near complete genome of BtNiLV1, and four conserved domains were predicted within the first two ORFs (Fig. 1b). Interestingly, the two large partially overlapping ORFs in the 5′-part of the genome characteristic of nidoviruses, were merged into one large read-through ORF in the predicted genome of BtNiLV1 (Fig. 1b). The polymerase of BtNiLV1 is related to Wuhan insect virus 19 (Accession: YP_009342322)[3], Fuefuki virus

(Accession: AWA82245)[4] and Wuhan nido-like virus 1 (Accession: YP_009345058)[3] with amino acid sequence identities of 32.8%, 30.7% and 30.5%, respectively. BtNiLV1 and these three related nido-like viruses were identified in different insect hosts, and cluster together to form a group in the phylogenetic tree in the order Nidovirales based on RdRP protein sequences (Fig. 2b).

A novel virus belonging to the family *Flaviviridae*, tentatively named Bemisia tabaci flavi-like virus 1 (BtFlLV1), was identified in *B. tabaci* ZJU-Q2. The predicted polyprotein ORF of BtFlLV1 has respectively 34.1% and 33.9% amino acid identity to the polyproteins of Bole tick virus 4 (BoTV4, Accession: YP_009179221)[5] and Trinbago virus (TrV, Accession: MN025505)[6] but the arrangement of the predicted conserved domains of BtFlLV1 is different to those of BoTV4 and TrV, and the coat protein was merely identified in BtFlLV1 as indicated in Fig. 1c. Phylogenetic analysis of the RdRP protein shows that BtFlLV1 apparently clusters within a distinct unclassified clade of viruses with invertebrate hosts more closely related to the genus *Pestivirus* (mammalian hosts), than to the other members of the family *Flaviviridae* (Fig. 2c), suggesting that this clade might represent a novel genus composed of ISVs as proposed in previous studies[5,6].

Negeviruses are a newly proposed group of ISVs in the order Martellivirales with a positive ssRNA genome of about 9 to 10kb[7]. We identified one negevirus (Bemisia tabaci negevirus 1, BtNeV1), three nege-like viruses (Bemisia tabaci nege-like virus 1, BtNeLV1; Bemisia tabaci nege-like virus 2, BtNeLV2; Bemisia tabaci nege-like virus 3, BtNeLV3) and two virga-like viruses (Bemisia tabaci virga-like virus 1, BtViLV1; Bemisia tabaci virga-like virus 2, BtViLV2) in the order Martellivirales from various whitefly datasets (Table 2). The two virga-like viruses with near complete genomes (BtViLV1, 10,187 nt; BtViLV2, 9535 nt) resembles the canonical organization in the family *Virgaviridae* and have domain signatures encoding for viral methyltransferase, peptidase, helicase, and RdRp in the predicted ORF1, and a capsid protein as ORF2 (Fig. 1e). Phylogenetic analysis shows that the newly identified nege-like and virga-like viruses clearly belong to two unclassified clades in the order Martellivirales distantly related to the established families currently recognized by ICTV (Fig. 2d). Although the 5′ terminus of BtNeV1, and the 3′ termini of BtNeLV2 and BtNeLV3 still need to be determined (Fig. 1d), a ML tree based on the conserved RdRP domain of nege/nege-like viruses indicates that BtNeV1 groups together with Loreto virus (Accession: NC_034158.1)[8] in the Negevirus clade, while the three novel

nege-like viruses (BtNeLV1, BtNeLV2, and BtNeLV3) constitute another unclassified clade between Negevirus and a plant virus family *Kitaviridae*. In addition, the two virga-like viruses are separated and grouped within two unclassified clades closely related to the family *Virgaviridae* (Fig. 2d).

Five novel picorna-like ssRNA viruses in the order Picornavirales were also identified in the datasets of *B. tabaci*, including a novel virus in the family *Dicistroviridae* (Bemisia tabaci dicistro-like virus 1, BtDiLV1), one novel virus in the family *Iflaviridae* (Bemisia tabaci iflavirus 1, BtIfV1), and another three novel viruses closely related to *Iflaviridae* in the order Picornavirales (Bemisia tabaci picorna-like virus 1, BtPiLV1; Bemisia tabaci picorna-like virus 2, BtPiLV2; Bemisia tabaci ifla-like virus 1, BtIfLV1) (Table 2). The predicted polyprotein of BtDiLV1 shares high similarity (84%) to Bundaberg bee virus 2 (Accession: MG995700)[9] and exhibits typical conserved dicistrovirus domain structure organization including helicase, peptidase, RdRP, and CP, whereas the other four ifla/ifla-related viruses, have the classical ifla-like virus structure with their structural protein (CP) at the N terminal and nonstructural proteins (helicase, protease, and RdRp) at the C terminal (Fig. 1f, g). An RdRP-based ML tree also shows that BtDiLV1 groups with viruses in the family of *Dicistroviridae*, and BtIfV1 clusters with the viruses in the family of *Iflaviridae*. However, although BtIfLV1 is closely related to iflaviruses, it is still hard to confirm whether or not it is a member of *Iflaviridae*, because the branch of BtIfLV1 is outside the core cluster of the other iflaviruses (Fig. 2e). Furthermore, BtPiLV1 and BtPiLV2 group together with several previously reported viruses and form a new unclassified cluster close to *Iflaviridae* but distinct from it and the other reported families in the order Picornavirales (Fig. 2e).

Positive-sense double-stranded RNA viruses

Two toti-like dsRNA viruses with nearly complete genomes were identified and tentatively named Bemisia tabaci toti-like virus 1 (BtToLV1) and Bemisia tabaci toti-like virus 2 (BtToLV2) (Table 2). BtToLV1 has a length of 6677 nt while the length of BtToLV2 is 7478 nt, and both viruses contains two ORFs encoding a proline-alanine rich protein (PArp) at the N terminal and RdRp at the C terminal similar to the reference totivirus Circulifer tenellus virus 1 (CiTV1, Accesion: NC_014360)[10]. However, while the two ORFs of previously reported totiviruses usually overlap, BtToLV1 and BtToLV2 have short intergenic regions between the two predicted ORFs

(Fig. 1h). Alignment of the predicted RArp and RdRP sequences indicated that BtToLV1 and BtToLV2 share 41.5% (PArp) and 44.9% (RdRp) identity. Phylogenetic trees based on the RdRp suggested that the newly identified BtToLV1 and BtToLV2 cluster within one of four unclassified clades (unclassified 1) in the family *Totiviridae*, and that this clade might represent a new genus in the family (Fig. 2f).

Segmented negative-sense single-stranded RNA viruses

Three closely related novel segmented negative ssRNA viruses belonging to the genus *Quaranjavirus* (family *Orthomyxoviridae*) were discovered with distinct cryptic species from different datasets of *B. tabaci* (Table 2), tentatively named Bemisia tabaci quaranjavirus 1 (BtQuV1), Bemisia tabaci quaranjavirus 2 (BtQuV2), and Bemisia tabaci quaranjavirus 3 (BtQuV3). These three novel quaranjaviruses share similarities to the nearly full-length segments of Mason Creek virus (MaCV) ORFs: Polymerase basic 1 protein (PB1, Accession: MK037473), Polymerase basic2 protein (PB2, Accession: MK037472), Polymerase Acidic protein (PA, Accession: MK037474), Nucleoprotein (NP, Accession: MK037475), and Hemagglutinin (HA, Accession: MK037476)[11] (Fig. 1i). We failed to identify a sixth segment Matrix protein (MP) for these new quaranjaviruses although the other five segments display relatively high abundance (coverage from 33X-519X, Table 2), possibly due to the low homology among the MPs of the genus *Quaranjavirus*[12,13]. The three quaranjaviruses of whitefly exhibit higher similarities to one another other in the nucleotide sequence of PB1 (77.4% - 88.8%) and the predicted RdRP (87.7% -98.6%) than they do MaCV (28%-56%) (Supplementary Figure 3 and Table 2). Phylogenetic analysis based on the conserved RdRP protein in the family *Orthomyxoviridae* clearly places the three novel quaranjaviruses of whitefly within the genus *Quaranjavirus*, forming one of the two subgroups in the genus together with MaCV, Longchuang virus[14], and Beihai orthomyxo-like virus 1[3] with strong bootstrap value support (Fig. 2g). Most quaranjaviruses were first identified from ticks and birds, but more recently several new quaranjaviruses have been identified in mosquitoes, flies, and spiders[2]. To the best of our knowledge, these are the first quaranjaviruses discovered from agricultural insect pests.

Diversity of other viral-like contigs in *B. tabaci*

Besides the twenty novel ISVs described above, we also identified several known and novel virus/viral-like contigs in *B. tabaci*. Thus, a new isolate of Potato virus S (PVS) (with coverage of 20X) was discovered in the whitefly dataset CU-B1 and tentatively named Potato virus S isolate Bt-MEAM1 (PVS-Bt-B) (Supplementary Fig. 3c). PVS, belonging to the genus *Carlavirus*, family *Betaflexiviridae,* is one of the most prevalent viruses in potato crops worldwide. The near complete genome sequence of PVS-Bt-B (7972 nt) was deposited in GenBank under the accession number of MW256706 and shares 99.3% nucleotide identity with the closest Potato virus S isolate NZ-O ab030 Lincoln (Accession: KU058656.1)[15]. It is of note that PVS is reported to be efficiently transmitted by aphids or plant-to-plant contact in a nonpersistent manner[16]. The relative abundance of PVS-Bt-B in whiteflies suggests another potential vector for PVS transmission in the field which needs further investigation.

Although only one plant virus was identified among the selected public whitefly datasets, which were mostly derived from stable lab populations, we discovered many diverse novel viral-like contigs in the whiteflies collected from cucumber in the field at Fuyang (sample FY-Q). This sample contained a total of six beny-like viruses (tentative named Bemisia tabaci beny-like virus 1-6, BtBeLV1-6) and six bromo-like viruses (Bemisia tabaci bromo-like virus 1-6, BtBromoLV1-6) with relatively high abundance (coverage) (Table 2). These viruses had relatively high similarities in their RdRp nucleotide sequences: 37.4-73.7% identity among the beny-like viruses and 36.8-87.5% among the bromo-like viruses (Supplementary Fig. 4). Conserved domain analysis indicated that all of the beny-like viruses have a similar conserved domain arrangement to the fungal virus Entomophthora benyvirus E (EnBVE, Accession: MK231113.1), and phylogenic analysis suggests that BtBeLV1-5 clustered together with EnBVE, as well as another two fungal viruses (Rhizoctonia solani beny-like virus1, RSBLV1 and Sclerotium rolfsii beny-like virus 1, SRBLV1). Interestingly, BtBeLV6 is different from the other beny-like viruses in whitefly and forms a clade with Hubei beny-like virus 1 closer to the clade of plant viruses (Supplementary Fig. 3a). Unexpectedly, BtBromoLV1-6 all have similar domain structures to a crab virus, Beihai charybdis crab virus 1 (BeCCV1, Accession: NC_032449.1)[3], although CP regions are missing in BtBromoLV4-6 perhaps because the 3'-termini are incomplete (Supplementary Fig. 3b). In phylogenic analysis, BtBromoLV1-6 all group together with BeCCV1, and form a unique unclassified clade separate from the plant viruses in the family *Bromoviridae* (Supplementary Fig.

3b). All of the identified novel ISVs and virus/viral-like sequences in the whitefly populations from the lab (NBU-B, NBU-Q) and field (FY-Q) were then verified and confirmed by RT-PCR, followed by Sanger sequencing.

Despite the fact that most of the viruses belonging to *Benyvirus* and *Bromoviridae* are plant viruses, BtBeLV1-5 clustered with the recently reported mycovirus EnBVE, and grouped with another clade of two fungal viruses (RSBLV1 and SRBLV1) (Supplementary Fig. 3a), suggesting the high possibility that BtBeLV1-5 are mycoviruses derived from the fungi that infect whitefly rather than being whitefly ISVs. It is common that mNGS can lead to the discovery of the viruses from the commensal microorganisms such as fungi and protozoa that infect hosts. Virome investigation and phylogenetic analysis in the cecal content of rabbits showed that the identified viruses were likely associated with the rabbit diet or the other commensal microorganisms such as fungi or protistz[17]. Similarly, two new dsRNA viruses (Spissistilus festinus virus 1 and Circulifer tenellus virus 1) isolated from two plant-feeding hemipteran insect pests were distantly related to the unclassified fungal dsRNA viruses Phlebiopsis gigantea virus 2 and Fusarium graminearum virus 3, and it remains unclear whether these viruses can replicate in insect cells or are merely associated microorganisms[10]. In our study, BtBromoLV1-6 from whitefly all group together in phylogenetic analysis with a crab virus BCCV1 and formed an unclassified clade clearly distinguished from all of the other plant virus clades in the family *Bromoviridae* (Supplementary Fig. 3b), strongly supporting the conclusion that viruses in this clade might be established as a new taxon of viruses in this family. It should be emphasized that the only field whitefly sample analyzed in this study had much more viral diversity (four novel ISVs, six novel beny-like viruses, and six bromo-like viruses) than most of the other whitefly datasets which are mostly derived from laboratory cultures, implying that the actual diversity of RNA viromes in field whitefly populations is still largely unexplored and will be worth investigating in the future.

1 Maes, P. *et al.* Taxonomy of the order Mononegavirales: second update 2018. *Arch Virol* **164**, 1233-1244 (2019).

2 Li, C. X. *et al.* Unprecedented genomic diversity of RNA viruses in arthropods reveals the ancestry of negative-sense RNA viruses. *elife* **4** (2015).

3 Shi, M. *et al.* Redefining the invertebrate RNA virosphere. *Nature* **540**, 539-543 (2016).

4 Medd, N. C. *et al.* The virome of *Drosophila suzukii*, an invasive pest of soft fruit. *Virus Evol* **4**,

vey009 (2018).
5 Shi, M. *et al.* Divergent Viruses Discovered in Arthropods and Vertebrates Revise the Evolutionary History of the Flaviviridae and Related Viruses. *J Virol* **90**, 659-669 (2016).
6 Sameroff, S. *et al.* Viral Diversity of Tick Species Parasitizing Cattle and Dogs in Trinidad and Tobago. *Sci Rep* **9**, 10421 (2019).
7 Vasilakis, N. *et al.* Negevirus: a proposed new taxon of insect-specific viruses with wide geographic distribution. *J Virol* **87**, 2475-2488 (2013).
8 Nunes, M. R. T. *et al.* Genetic characterization, molecular epidemiology, and phylogenetic relationships of insect-specific viruses in the taxon Negevirus. *Virology* **504**, 152-167 (2017).
9 Roberts, J. M. K., Anderson, D. L. & Durr, P. A. Metagenomic analysis of Varroa-free Australian honey bees (Apis mellifera) shows a diverse Picornavirales virome. *J Gen Virol* **99**, 818-826 (2018).
10 Spear, A., Sisterson, M. S., Yokomi, R. & Stenger, D. C. Plant-feeding insects harbor double-stranded RNA viruses encoding a novel proline-alanine rich protein and a polymerase distantly related to that of fungal viruses. *Virology* **404**, 304-311 (2010).
11 Walker, P. J. *et al.* Characterization of Three Novel Viruses from the Families Nyamiviridae, Orthomyxoviridae, and Peribunyaviridae, Isolated from Dead Birds Collected during West Nile Virus Surveillance in Harris County, Texas. *Viruses* **11** (2019).
12 Allison, A. B. *et al.* Cyclic avian mass mortality in the northeastern United States is associated with a novel orthomyxovirus. *J Virol* **89**, 1389-1403 (2015).
13 Cholleti, H. *et al.* Viral metagenomics reveals the presence of highly divergent quaranjavirus in Rhipicephalus ticks from Mozambique. *Infect Ecol Epidemiol* **8**, 1478585 (2018).
14 Zhang, X. L., Z.; Tan, Q.; Zhang, H.; Zhou, H.; Li, B.; Wu, D.; Cao, Y.; Wang, H.; Liang, G.;. Identification and Molecular Characterization of a Novel Virus of the Family Orthomyxoviridae (Longchuan Virus). *Chinese Journal of Virology* **35**, 599-605 (2019).
15 Blouin, A. G. *et al.* A new virus discovered by immunocapture of double-stranded RNA, a rapid method for virus enrichment in metagenomic studies. *Mol Ecol Resour* **16**, 1255-1263 (2016).
16 Santillan, F. W. *et al.* The Biology and Phylogenetics of Potato virus S Isolates from the Andean Region of South America. *Plant Dis* **102**, 869-885 (2018).
17 Mahar, J. E., Shi, M., Hall, R. N., Strive, T. & Holmes, E. C. Comparative Analysis of RNA Virome Composition in Rabbits and Associated Ectoparasites. *J Virol* **94** (2020).

Supplementary Table 1. Primers used for RT-PCR and qRT-PCR

Newly identified viruses in whitefly	Forward primer (5’-3’)	Reverse primer (5’-3’)
**Primers used for RT-PCR**		
Bemisia tabaci arlivirus 2_segment1	TTGTAATTCAGGGGGTTAGGGG	ATTTGGAAGGTTGCTTCTGTAC
Bemisia tabaci arlivirus 2_segment2	ATGGATGATCAAGACTATGATC	CATCCAAAAGAGATAATAGTGG
Bemisia tabaci picorna-like virus 1_segment1	ATGGCTACTACAGGTTATGAAG	TCACGATCAGCTAAATCATTAC
Bemisia tabaci picorna-like virus 1_segment2	TCACGATCAGCTAAATCATTAC	TTGATGTGCAACAGTATAAGGC
Bemisia tabaci virga-like virus 2_segment1	ACAGAGATAACATCCGCTATAG	AGGGCGATCCTCTCGATTAATG
Bemisia tabaci virga-like virus 2_segment2	ACGTTCTCACCGGCGAGAAAAG	CATCATGTTGCTCAGCAGCTTG
Bemisia tabaci arlivirus 1_segment1	GAGGTCCTTTGACATTTTCGTC	CATCTTGTCTTCTGTTCGGGAG
Bemisia tabaci arlivirus 1_segment2	TGAGCGTTTTCTCTGGAGCTAG	TGACCAAAATGCCTGTAGATTC
Bemisia tabaci virga-like virus 1_segment1	GAGATGTTTTTGAACGCGACGC	CTCACTTAAATAACACCTGCC
Bemisia tabaci virga-like virus 1_segment2	CGTGTTGCCGATTTTTTCAACC	TTTCTCAATCGCTCTGTCAGGC
Bemisia tabaci toti-like virus 1_segment1	GAAGTTGACAGTCAGACATTCG	ACGAAACCTGCGAAAGTTGCAG
Bemisia tabaci toti-like virus 1_segment2	GCTACTGCATCTGAAAAAATGG	ATTCAGCGGTGTGGGATAATAC
Bemisia tabaci Quaranja-like virus 1_segment1	CTGCACAAGGTTTTGGTAAGAC	GCTCCTCGTTGAACTCCACATC

Bemisia tabaci Quaranja-like virus 1_segment2	ATCCATTACCACAATTGCTATG	CCACTCCTTACATTACTCCTG
Bemisia tabaci pesti-like virus 1	CTTGTCTGTCTCCCCGTTGTAC	TGCAGATTGGAGAGAAACTCCG
Bemisia tabaci dicistro-like virus 1	GTGCAATCACGTCTGGGTGATC	CGGAGTGTGTGTCCTAGGATAC
Bemisia tabaci nege-like virus 1	AAAACCGATGAAGAATCGGTTC	GGATTAAGAGATGAGACGTTAAC
Bemisia tabaci nege-like virus 2	GTCTTCCCCCAGAAGCCCGTCA	CATAAACATTGCCCTTGGGGAT
Bemisia tabaci nege-like virus 3	AGTGATCCGGATGTTAACTCGG	TCACGATTGACCAATTTATGTG
Bemisia tabaci bromo-like virus 1	TCGGAACCTTCTCAAGCCTCC	TGATGTGGAGCAGCGGCGTTAG
Bemisia tabaci bromo-like virus 2	CTTCCGAATCTTCTCAGGTCGC	GTAGGCAGGAACGGAGACAAGG
Bemisia tabaci bromo-like virus 3	CTTCTTCCCTTTCCAATGATCC	GAGGGAGTCAGGGGCCAGAAAG
Bemisia tabaci bromo-like virus 4	CGCTTACTCGTTCTGCTAT	TTAGTTGGTGCTGTATCTCC
Bemisia tabaci bromo-like virus 5	TTGGATACCGATGTGACTC	GCGTTCTACCACTATGTTG
Bemisia tabaci bromo-like virus 6	TCCGACAACGCCATATAC	GTAACGACAGTAGATAGACAG
Bemisia tabaci beny-like virus 1	ACGTCAACTCATCGGCTGTCAC	GCAGCCAAGGTGCGCATTAGT
Bemisia tabaci beny-like virus 2	TGTGAACACGTCTTCCGCAGTC	GCGGCGAGTGTTCTCATCAGT
Bemisia tabaci beny-like virus 3	AATGGCGCCCAGAGAAGTTGTC	CCAACGACCGCAGCAACATTTC
Bemisia tabaci beny-like virus 4	GTTACGAGGAATACTACGATTG	AACGGCTGGAACGATTAC
Bemisia tabaci beny-like virus 5	TGCCTCAGTCCAAGTATTC	TCCAACAGACACGCTAAG

Bemisia tabaci beny-like virus 6	ACTCCGACACTCTTAACTATC	CGTCCATCCTAATGTTCTCA
**Primers used for qRT-PCR**		
Bemisia tabaci arlivirus 2	GTCCTAATGATTGCTGATGTAG	ATATTCTGTGCTGAGTGGTAG
Bemisia tabaci picorna-like virus 1	GCCTACAACTACTACAACAGT	CTACTAACATCAAGCCATAACC
Bemisia tabaci virga-like virus 2	AACCTCGCTAGTATTCCTAAG	GATTCACGATGTCTGGATATG
Bemisia tabaci virga-like virus 1	GGAACGAATGAATGAGTTGAG	CAGAACGCCTAATACAGATAAC
Bemisia tabaci Quaranja-like virus 1	GAGGTAGAACGACAACTGATT	TAGGTCCATCATACCGTAGTT
Bemisia tabaci 18s RNA	CGGAAGGATTGACAGATTGA	TCTCGTTCGTTATCGGAATT

Supplementary Table 2. Details of sequences obtained from GenBank for the construction of phylogeny tree

Virus name	GenBank ID	Year	Host species	Reference
Arlivirus				
Mokola lyssavirus	YP_142354	1997	cat	(Le Mercier et al. 1997)
Aravan lyssavirus	YP_007641396	2008	mouse	(Kuzmin et al. 2008)
Odonate arlivirus	YP_009336924	2016	dragonflies, damselflies	(Shi et al. 2016a)
Gerrid arlivirus	YP_009288955	2015	Gerridae	(Li et al. 2015)
Lishi arlivirus	YP_009303699	2015	Araneae	(Li et al. 2015)
Wuchang arlivirus	YP_009342285	2016	*Blattella germanica*	(Shi et al. 2016a)
Amsterdam virus	QEQ50497	2019	*Mus musculus*	(Williams et al. 2019)
Tacheng arlivirus	YP_009304420	2015	*Argas miniatus*	(Li et al. 2015)
Lampyris noctiluca rhabdo-like virus 1	QBP37026	2019	*Lampyris noctiluca*	(Viljakainen et al. 2020)
Hubei rhabdo-like virus 3	YP_009336889	2016	Coleoptera mix	(Shi et al. 2016a)
Linepithema humile rhabdo-like virus 1	AXA52562	2018	Argentine ant	(Viljakainen et al. 2018)
Berant virus	AOC55081	2016	--	--
**Nidovirales**				
Equine arteritis virus	NP_705590	2020	equine	(Ziebuhr et al. 2000)
xinzhou toro-like virus	YP_009344970.1	2016	Snake-associated nematodes	(Shi et al. 2016a)
Wuhan insect virus 19	YP_009342322	2016	Insect mix	(Shi et al. 2016a)
Fuefuki virus	AWA82245	2018	*Drosophila suzukii*	(Medd et al. 2018)
Bat coronavius	YP_009824989.2	2018	*Eidolon helvum*	(Yinda et al. 2018)
Casuarina virus	YP_009026378	2014	*Coquillettidia xanthogaster*	(Warrilow et al. 2014)
Beihai nido-like virus 1	YP_009333346	2016	*Turritella sea snails*	(Shi et al. 2016a)
Beihai nido-like virus 2	APG77322	2016	Charybdis crab mix	(Shi et al. 2016a)
Gill-associated virus	YP_001661455	2002	*Penaeus monodon*	(Cowley et al. 2002)
Flaviviridae				
Xinzhou spider virus 3	YP_009254746	2015	spider	(Shi et al. 2016b)

Shayang spider virus 4	YP_009179219	2015	*Neoscona nautica*	(Shi et al. 2016b)
Wuhan centipede virus	YP_009254745	2015	*Scolopocryptops*	(Shi et al. 2016b)
Shuangao lacewing virus 2	YP_009179223	2015	Chrysopidae	(Shi et al. 2016b)
Culex tritaeniorhynchus flavi-like virus	BBQ05092	2020	*Culex vishnui*	(Faizah et al. 2020)
Beihai barnacle viurs 1	YP_009179226	2015	*Amphibalanus rhizophorae*	(Shi et al. 2016a)
Trinbago virus	QDW81037	2019	ticks	(Sameroff et al. 2019)
Bole tick virus 4	YP_009179221	2015	*Hyalomma asiaticum*	(Shi et al. 2016a)
porcine pestivirus 1	YP_009508311	2015	porcine	(Hause et al. 2015)
pestivirus J	YP_009109567	2014	*Rattus norvegicus*	(Firth et al. 2014)
Porcine pestivirus	QGW35992	2020	pig	(Zhou et al. 2019)
Pronghom antelope pestivirus	YP_009026415	2014	pronghorn antelope	(Neill et al. 2014)
Bovine viral diarrhea virus 2	AZQ00665	2019	cattle	(Neill et al. 2019)
pestivirus giraffe-1 H138	NP_620053	2001	*Giraffa camelopardalis*	(Avalos-Ramirez et al. 2001)
Border disease virus X818	NP_620062	1998	sheep	(Becher et al. 1998)
Hog cholera virus	BAA08476	1995	hog	(Ishikawa et al. 1995)
pestivirus strain aydin/04-TR	YP_006860588	2012	sheep	(Becher et al. 2012)
**Martellivirales**				
Tomato mottle mosaic virus	YP_008492928	2013	tomato	(Li et al. 2013)
Blueberry necrotic ring blotch virus	YP_004901701	2013	blueberry	(Quito-Avila et al. 2013)
Nephila clavipes virus 4	YP_009552461.1	2017	*Nephila clavipes*	(Debat 2017)
Citrus leprosis virus C2	YP_009509062	2013	sweet orange	(Roy et al. 2013)
Hibiscus green spot virus 2	YP_004928118	2012	*Citrus volkameriana*	(Melzer et al. 2012)
Nephila clavipes virus 3	YP_009552459	2017	*Nephila clavipes*	(Debat 2017)
Loreto virus	YP_009351835.1	2017	*Anopheles albimanus*	(Nunes et al. 2017)
Big Cypress virus	YP_009351821	2017	*Anopheles atropos*	(Nunes et al. 2017)
Ying Kou virus	YP_009552739	2019	*Culex pipiens pallens*	--
Negevirus Nona 1	BAS69360	2016	*Aedes*	(Kawakami et al. 2016)
Daeseongdong virus 1	YP_009182191	2016	*Culex bitaeniorhynchus, Culex pipiens*	(Hang et al. 2016)

Piura virus	YP_009351830	2017	*Culex* sp.	(Nunes et al. 2017)
Castlerea virus	YP_009362298	2017	*Anopheles, Culex*, *Aedes*	(O'Brien et al. 2017)
West Accra virus	BBN20799.1	2015	*Aedes aegypti*	(Amoa-Bosompem et al. 2020)
Manglie virus	QBR99594.1	2011	pooled mosquitos	--
Negev virus	YP_009256205	2017	*Culex vishunui*	(Fujita et al. 2017)
Hubei virga-like virus 1	YP_009337423.1	2013	Mosquitoes	(Shi et al. 2016a)
Atrato virga-like virus 1	QHA33734	2020	*Culex sp.*	-
Hubei virga-like virus 2	YP_009337412	2016	*Odonata mix Hubei*	(Shi et al. 2016a)
Atrato virga-like virus 3	QHA33742	2020	*Mansonia titillans*	--
Adelphocoris suturalis virus	YP_009336476	2015	*Adelphocoris suturalis*	(Li et al. 2017)
Megastigmus ssRNA virus	QDZ71189.1	2011	*Megastigmus spermotrophus*	--
Dougjudy virga-like virus	QIJ70136	2020	*Calliphora vicina*	(Mahar et al. 2020)
Hubei virga-like virus 10	APG77802	2016	*House centipede* mix	(Shi et al. 2016a)
Abisko virus	YP_009408586	2017	*Epirrita autumnata*	(De Miranda et al. 2017)
**Picornavirales**				
Solenopsis invicta virus 1	YP_164440	2004	fire ants	(Valles et al. 2004)
Acute bee paralysis virus	NP_066241	2000	*Apis mellifera*	(Govan et al. 2000)
Israeli acute paralysis virus	YP_001040002	2007	dead bees	(Maori et al. 2007)
Kashmir bee virus	NP_851403	2004	Kashmir bee	(de Miranda et al. 2004)
Human blood-associated dicistrovirus	AWK23470	2018	human serum	(Phan et al. 2018)
Weivirus-like virus sp.	QJI53767.1	2018	*Phylloscopus*	--
Bundaberg bee virus 2	AWK77852	2018	*Apis mellifera*	(Roberts et al. 2018)
Mud crab virus	YP_004063985	2010	*Scylla serrata*	(Guo et al. 2013)
Taura syndrome virus	NP_149057	2002	*Penaeus vannamei*	(Mari et al. 2002)
Drosophlia C virus	NP_044945	1998	*Drosophila*	(Johnson and Christian 1998)
Cricket paralysis virus	NP_647481	2000	crickets	(Wilson et al. 2000)
Homalodisca coagulate virus	YP_610950	2005	*Homalodisca coagulata*	(Hunnicutt et al. 2006)
Triatoma virus	NP_620562	2000	*Triatoma*	(Czibener et al. 2000)

Black queen cell virus	NP_620564	2000	*Apis mellifera*	(Leat et al. 2000)
Plautia stali intestine virus	NP_620555	1998	*Plautia stali*	(Sasaki et al. 1998)
Himetobi P virus	NP_620560	1999	*Laodelphax striatellus*	(Nakashima et al. 1999)
Tomato matilda virus	QDW80878	2019	tomato	--
Bundaberg bee virus 2	AWK77852	2018	*Apis mellifera*	(Roberts et al. 2018)
Lygus lineolaris virus 1	YP_009505598	2012	*Lygus lineolaris*	(Perera et al. 2012)
Sacbrood virus	NP_049374	1999	*Apis mellifera*	(Ghosh et al. 1999)
Sanxia water strider virus 8	YP_009336688	2016	water striders	(Shi et al. 2016a)
Harmonia axyridis virus 1	QFR15911	2018	*Harmonia axyridis*	--
Hubei picorna-like virus 27	YP_009337161	2016	*Coleoptera mix*	(Shi et al. 2016a)
Hubei picorna-like virus 28	YP_009337284	2016	Odonata mix	(Shi et al. 2016a)
Hubei picorna-like virus 31	YP_009330055	2016	Odonata mix	(Shi et al. 2016a)
Hubei picorna-like virus 40	YP_009336539	2016	*House centipede mix*	(Shi et al. 2016a)
Hubei tetragnatha maxillosa virus 2	YP_009337271	2016	Arthropod mix	(Shi et al. 2016a)
Slow bee paralysis virus	YP_003622540	2010	*Apis mellifera*	(de Miranda et al. 2010)
Nilaparvata lugens honeydew virus 1	YP_009505599	2013	*Nilaparvata lugens*	(Murakami et al. 2013)
Bombyx mori iflavirus	YP_009162630	2015	*Bombyx mori*	(Suzuki et al. 2015)
Heliconius erato iflavirus	YP_009026409	2014	*Heliconius erato*	(Smith et al. 2014)
Lymantria dispar iflavirus 1	YP_009047245	2014	*Lymantria dispar*	(Carrillo-Tripp et al. 2014)
Antheraea pernyi iflavirus	YP_009002581	2014	*Antheraea pernyi*	(Geng et al. 2014)
Darwin bee virus 3	AWK77848	2018	*Apis mellifera*	(Roberts et al. 2018)
Deformed wing virus	NP_853560.2	2006	*Apis mellifera*	(Lanzi et al. 2006)
Varroa destructor virus 1	YP_145791	2004	*Varroa destructor*	(Ongus et al. 2004)
Varroa destructor virus 2	QFS19921	2019	*Varroa destructor*	(Herrero et al. 2019)
Yonago Culex iflavirus	BBQ04784	2020	*Culex vishnui*	(Faizah et al. 2020)
Watson virus	QED21508	2019	Australian fleas	(Harvey et al. 2019)
Lampyris noctiluca iflavirus 2	QBP37020	2017	*Lampyris noctiluca*	(Viljakainen et al. 2020)
Diaphorina citri picorna-like virus	ALJ52073	2014	*Diaphorina citri*	(Nouri et al. 2016)
Brevicoryne brassicae virus	YP_001285409	2007	*Brevicoryne brassicae*	(Ryabov 2007)
Nephila clavipes virus 1	YP_009553501	2007	*Brevicoryne brassicae*	(Ryabov 2007)

Lysiphlebus fabarum rna-virus	QFP98431	2019	*Lysiphlebus fabarum*	(Lüthi et al. 2020)
Dinocampus coccinellae paralysis virus	YP_009111311	2015	parasitic wasp	(Dheilly et al. 2015)
Tesano Aedes virus	BBN21000	2020	Mosquito	(Amoa-Bosompem et al. 2020)
Orhomyxoviridae				
Tilapia lake virus	QJD15208	2020	Fish	(Thawornwattana et al. 2020)
Thogoto virus	YP_145810	1999	mammalian	(Weber et al. 1999)
Dhori virus	YP_009352882	2001	mammalian	--
Influenza C virus	YP_089654	2004	bird	(Muraki et al. 2004)
Influenza D virus	YP_009449560	2013	pig	(Hause et al. 2013)
Influenza A virus	NP_040987	1982	bird	(Fields and Winter 1982)
Influenza B virus	NP_056657	1986	bird	(Kemdirim et al. 1986)
Tjuloc virus	AFN73049	2013	--	--
Quaranfil quaranjavirus	AXL67889	2009	Ticks 、Birds	(Presti et al. 2009)
Johnston Atoll virus	YP_009665204	2009	Ticks 、Birds	(Presti et al. 2009)
Wellfleet Bay virus	YP_009110687	2015	Birds	(Allison et al. 2015)
Araguari virus	ASR92125	2016	--	--
Uumaja virus	QKK82920	2020	Ticks	(Pettersson et al. 2020)
Zambezi tick virus 1	AWU49720	2018	Ticks	(Cholleti et al. 2018)
Beihai orthomyxo-like virus 1	AWU49720	2018	Ticks	(Cholleti et al. 2018)
Longchuang virus	QKK82920	2020	Ticks	(Pettersson et al. 2020)
Mason Creek virus	QFQ60710	2019	Birds	(Walker et al. 2019)
Totiviridae				
Murri virus	QHA33714	2020	*Coquillettidia venezuelensis*	--
dsRNA virus environmental sample	AJT39599	2015	Mosquitoes	(Chandler et al. 2015)
Persimmon latent virus	YP_009025166	2013	*Diospyros kaki*	(Ito et al. 2013)
Circulifer tenellus virus 1	YP_003800003	2009	*Circulifer tenellus*	(Spear et al. 2010)
**Beny-like virus**				
Helicoverpa armigera stunt virus	NP_049235.1	1995	*Helicoverpa armigera*	(Hanzlik et al. 1995)
Hubei Beny-like virus 1	QED42938.1	2016	Diptera mix	(Shi et al. 2016a)

Rice stripe necrosis virus	QNU12832.1	2007	*Oryza sativa*	(Lozano and Morales 2009)
Beet necrotic yellow vein virus	NP_705701.1	1996	*Tetragonia expansa*	(Saito et al. 1996)
Beet soil-borne mosaic virus	AAF91435.1	2006	*Beta vulgaris*	(D'Alonzo et al. 2008)
Monilinia benyvirus C	QED42944	2018	--	--
Sclerotium rolfsii beny-like virus 1	AZF86092.1	2016	*Athelia rolfsii*	--
Rhizoctonia solani beny-like virus 1	QDW81308.1	2013	*Rhizoctonia solani beny-like virus 1*	(Picarelli et al. 2019)
Entomophthora benyvirus E	QED42962	2016	*Entomophthora muscae*	--
**Bromo-like virus**				
Pelargonium zonate spot virus	BBA22646.1	2003	*Nicotiana glutinosa*	(Finetti-Sialer and Gallitelli 2003)
Cucumber mosaic virus	NP049324.1	1982	cucumber	(Gould and Symons 1982)
Peanut stunt virus	NP049332.1	2012	*Pisum sativum*	(Obrępalska-Stęplowska et al. 2012)
Cowpea chlorotic mottle virus	NP613275.1	1982	cowpea	(Dasgupta and Kaesberg 1982)
Tomato aspermy virus	NP620761.1	1994	tomato	(Salánki et al. 1994)
Spring beauty latent virus	NP689393.1	2003	*Arabidopsis thaliana*	(Fujisaki et al. 2003)
Pineapple mealybug wilt-associated virus 1	YP001642337.1	2008	pineapple	(Melzer et al. 2008)
Gayfeather mild mottle virus	YP002640501.1	2009	*Melandrium yellow fleck virus*	(Adams et al. 2009)
Melandrium yellow fleck virus	YP003208108.1	2018	*Silene latifolia*	(Minicka et al. 2020)
Grapevine leafroll-associated virus 5	YP004901687.1	2012	*Vitis vinifera*	(Thompson et al. 2012)
Amazon lily mild mottle virus	YP006576519.1	2019	*Paeonia lactiflora*	(Thomas-Sharma et al. 2018)
Blueberry shock virus	YP008519305.1	2015	blueberry	(Thomas-Sharma et al. 2018)
Beihai charybdis crab virus 1	YP009333242.1	2016	crab	(Shi et al. 2016a)
Sambucus virus S	YP009551509.1	2015	*Sambucus nigra*	(Šafářová et al. 2018)
Tea plant line pattern virus	YP009551568.1	2019	*Camellia sinensis*	--
Apple necrotic mosaic virus	YP009551615.1	2013	apple	(Xing et al. 2018)
Alfalfa mosaic virus	YP053235.1	1984	tobacco	(Joshi et al. 1984)
Cassia yellow blotch virus	YP233102.2	2005	Cassia	(Iwahashi et al. 2005)

Isolate Potato virus S

Blueberry scorch virus	AAY18403.1	2017	*Sambucus nigra*	--
Potato virus S	AMN08810.1	1995	*Solanum tuberosum*	(Zavriev et al. 1991)
Opuntia virus H	ANH10854.2	2015	*Opuntia ficus*	--
Kalanchoe latent virus	APX54977.1	2006	*Chenopodium quinoa*	(Dinesen et al. 2009)
Passiflora latent virus	AXL95764.1	2013	*Passiflora edulis*	(Jover-Gil et al. 2018)
Lily symptomless virus	QBS17038.1	2009	Lilium	(Xu et al. 2011)
Aconitum latent virus	Quinvirinae	2002	Delphinium	(Fuji et al. 2002)
Cole latent virus	Quinvirinae	2015	*Brassica oleracea*	(Belintani et al. 2002)
Pea streak virus	Quinvirinae	2008	*Medicago sativa*	--
Apple stem pitting virus	Quinvirinae	1994	apple	(Jelkmann 1994)
Grapevine virus T	Quinvirinae	2018	*Vitis vinifera*	(Diaz-Lara et al. 2020)
Ligustrum necrotic ringspot virus	YP001661446.1	2011	*Phlox stolonifera*	
Hydrangea chlorotic mottle virus	YP002985636.1	2007	*Hydrangea macrophylla*	(Tang et al. 2010)
Mirabilis jalapa mottle virus	YP004901680.1	2010	*Mirabilis jalapa*	(Hatlestad et al. 2011)

Adams, IP, Glover, RH, Monger, WA, Mumford, R, Jackeviciene, E, Navalinskiene, M, Samuitiene, M and Boonham, N (2009) Next-generation sequencing and metagenomic analysis: a universal diagnostic tool in plant virology. *Molecular plant pathology* **10**: 537-545.

Allison, AB, Ballard, JR, Tesh, RB, Brown, JD, Ruder, MG, Keel, MK, Munk, BA, Mickley, RM, Gibbs, SE and da Rosa, APT (2015) Cyclic avian mass mortality in the northeastern United States is associated with a novel orthomyxovirus. *Journal of virology* **89**: 1389-1403.

Amoa-Bosompem, M, Kobayashi, D, Murota, K, Faizah, AN, Itokawa, K, Fujita, R, Osei, JHN, Agbosu, E, Pratt, D and Kimura, S (2020) Entomological Assessment of the Status and Risk of Mosquito-borne Arboviral Transmission in Ghana. *Viruses* **12**: 147.

Avalos-Ramirez, R, Orlich, M, Thiel, H-J and Becher, P (2001) Evidence for the presence of two novel pestivirus species. *Virology* **286**: 456-465.

Becher, P, Orlich, M and Thiel, H-J (1998) Complete genomic sequence of border disease virus, a pestivirus from sheep. *Journal of virology* **72**: 5165-5173.

Becher, P, Schmeiser, S, Oguzoglu, TC and Postel, A (2012) Complete genome sequence of a novel pestivirus from sheep. In: *Book Complete genome sequence of a novel pestivirus from sheep* (Editor, ed.^eds.). Vol., pp. Am Soc Microbiol, City.

Belintani, P, Gaspar, J, Targon, M and Machado, M (2002) Evidence supporting the recognition of Cole latent virus as a distinct Carlavirus. *Journal of Phytopathology* **150**: 330-333.

Carrillo-Tripp, J, Krueger, EN, Harrison, RL, Toth, AL, Miller, WA and Bonning, BC (2014) Lymantria dispar iflavirus 1 (LdIV1), a new model to study iflaviral persistence in lepidopterans.

Journal of General Virology **95**: 2285-2296.

Chandler, JA, Liu, RM and Bennett, SN (2015) RNA shotgun metagenomic sequencing of northern California (USA) mosquitoes uncovers viruses, bacteria, and fungi. *Frontiers in microbiology* **6**: 185.

Cholleti, H, Hayer, J, Mulandane, FC, Falk, K, Fafetine, J, Berg, M and Blomström, A-L (2018) Viral metagenomics reveals the presence of highly divergent quaranjavirus in Rhipicephalus ticks from Mozambique. *Infection ecology & epidemiology* **8**: 1478585.

Cowley, JA, Dimmock, CM and Walker, PJ (2002) Gill-associated nidovirus of Penaeus monodon prawns transcribes 3′-coterminal subgenomic mRNAs that do not possess 5′-leader sequences. *Journal of General Virology* **83**: 927-935.

Czibener, C, La Torre, JL, Muscio, OA, Ugalde, RA and Scodeller, EA (2000) Nucleotide sequence analysis of Triatoma virus shows that it is a member of a novel group of insect RNA viruses. *Journal of General Virology* **81**: 1149-1154.

D'Alonzo, M, Bianchi, L, Autonell, C, Gilmer, D and Ratti, C (2008) New Beet soil-borne mosaic virus RNA-4 form isolation from infected sugar beet roots. In: *Book New Beet soil-borne mosaic virus RNA-4 form isolation from infected sugar beet roots* (Editor, ed.^eds.). Vol., pp. 49-53. Julius Kühn-Institute, Federal Research Centre for Cultivated Plants, City.

Dasgupta, R and Kaesberg, P (1982) Complete nucleotide sequences of the coat protein messenger RNAs of brome mosaic virus and cowpea chlorotic mottle virus. *Nucleic Acids Research* **10**: 703-713.

de Miranda, JR, Dainat, B, Locke, B, Cordoni, G, Berthoud, H, Gauthier, L, Neumann, P, Budge, GE, Ball, BV and Stoltz, DB (2010) Genetic characterization of slow bee paralysis virus of the honeybee (Apis mellifera L.). *Journal of General Virology* **91**: 2524-2530.

de Miranda, JR, Drebot, M, Tyler, S, Shen, M, Cameron, CE, Stoltz, D and Camazine, S (2004) Complete nucleotide sequence of Kashmir bee virus and comparison with acute bee paralysis virus. *Journal of General Virology* **85**: 2263-2270.

De Miranda, JR, Hedman, H, Onorati, P, Stephan, J, Karlberg, O, Bylund, H and Terenius, O (2017) Characterization of a novel RNA virus discovered in the autumnal moth Epirrita autumnata in Sweden. *Viruses* **9**: 214.

Debat, HJ (2017) An RNA virome associated to the golden orb-weaver spider Nephila clavipes. *Frontiers in microbiology* **8**: 2097.

Dheilly, NM, Maure, F, Ravallec, M, Galinier, R, Doyon, J, Duval, D, Leger, L, Volkoff, A-N, Missé, D and Nidelet, S (2015) Who is the puppet master? Replication of a parasitic wasp-associated virus correlates with host behaviour manipulation. *Proceedings of the Royal Society B: Biological Sciences* **282**: 20142773.

Diaz-Lara, A, Golino, D, Preece, JE and Al Rwahnih, M (2020) Development of RT-PCR degenerate primers to overcome the high genetic diversity of grapevine virus T. *Journal of Virological Methods*: 113883.

Dinesen, M, Lundmark, M and Albrechtsen, M (2009) Complete genome sequences of two isolates of Kalanchoë latent virus. *Archives of virology* **154**: 1173-1175.

Faizah, AN, Kobayashi, D, Isawa, H, Amoa-Bosompem, M, Murota, K, Higa, Y, Futami, K, Shimada, S, Kim, KS and Itokawa, K (2020) Deciphering the Virome of Culex vishnui Subgroup Mosquitoes, the Major Vectors of Japanese Encephalitis, in Japan. *Viruses* **12**: 264.

Fields, S and Winter, G (1982) Nucleotide sequences of influenza virus segments 1 and 3 reveal

mosaic structure of a small viral RNA segment. *Cell* **28**: 303-313.

Finetti-Sialer, M and Gallitelli, D (2003) Complete nucleotide sequence of Pelargonium zonate spot virus and its relationship with the family Bromoviridae. *Journal of General Virology* **84**: 3143-3151.

Firth, C, Bhat, M, Firth, MA, Williams, SH, Frye, MJ, Simmonds, P, Conte, JM, Ng, J, Garcia, J and Bhuva, NP (2014) Detection of zoonotic pathogens and characterization of novel viruses carried by commensal Rattus norvegicus in New York City. *MBio* **5**.

Fuji, S, Yamamoto, H, Inoue, M, Yamashita, K, Fukui, Y, Furuya, H and Naito, H (2002) Complete nucleotide sequence of the genomic RNA of Aconitum latent virus (genus Carlavirus) isolated from Delphinium sp. *Archives of virology* **147**: 865-870.

Fujisaki, K, Hagihara, F, Kaido, M, Mise, K and Okuno, T (2003) Complete nucleotide sequence of spring beauty latent virus, a bromovirus infectious to Arabidopsis thaliana. *Archives of virology* **148**: 165-175.

Fujita, R, Kuwata, R, Kobayashi, D, Bertuso, AG, Isawa, H and Sawabe, K (2017) Bustos virus, a new member of the negevirus group isolated from a Mansonia mosquito in the Philippines. *Archives of virology* **162**: 79-88.

Geng, P, Li, W, Lin, L, de Miranda, JR, Emrich, S, An, L and Terenius, O (2014) Genetic characterization of a novel iflavirus associated with vomiting disease in the chinese oak silkmoth Antheraea pernyi. *PLoS One* **9**: e92107.

Ghosh, R, Ball, B, Willcocks, M and Carter, M (1999) The nucleotide sequence of sacbrood virus of the honey bee: an insect picorna-like virus. *Journal of General Virology* **80**: 1541-1549.

Gould, AR and Symons, RH (1982) Cucumber mosaic virus RNA 3: Determination of the nucleotide sequence provides the amino acid sequences of protein 3A and viral coat protein. *European Journal of Biochemistry* **126**: 217-226.

Govan, V, Leat, N, Allsopp, M and Davison, S (2000) Analysis of the complete genome sequence of acute bee paralysis virus shows that it belongs to the novel group of insect-infecting RNA viruses. *Virology* **277**: 457-463.

Guo, Z-X, He, J-G, Xu, H-D and Weng, S-P (2013) Pathogenicity and complete genome sequence analysis of the mud crab dicistrovirus-1. *Virus research* **171**: 8-14.

Hang, J, Klein, TA, Kim, H-C, Yang, Y, Jima, DD, Richardson, JH and Jarman, RG (2016) Genome sequences of five arboviruses in field-captured mosquitoes in a unique rural environment of South Korea. *Genome announcements* **4**.

Hanzlik, TN, Dorrian, SJ, Johnson, KN, Brooks, EM and Gordon, KH (1995) Sequence of RNA2 of the Helicoverpa armigera stunt virus (Tetraviridae) and bacterial expression of its genes. *Journal of General Virology* **76**: 799-811.

Harvey, E, Rose, K, Eden, J-S, Lawrence, A, Doggett, SL and Holmes, EC (2019) Identification of diverse arthropod associated viruses in native Australian fleas. *Virology* **535**: 189-199.

Hatlestad, GJ, Elam, L, Gonzalez, A and Lloyd, AM (2011) Mirabilis jalapa mottle virus: a new carlavirus infecting four o'clocks. *Archives of virology* **156**: 2109.

Hause, BM, Collin, EA, Peddireddi, L, Yuan, F, Chen, Z, Hesse, RA, Gauger, PC, Clement, T, Fang, Y and Anderson, G (2015) Discovery of a novel putative atypical porcine pestivirus in pigs in the USA. *Journal of General Virology* **96**: 2994-2998.

Hause, BM, Ducatez, M, Collin, EA, Ran, Z, Liu, R, Sheng, Z, Armien, A, Kaplan, B, Chakravarty, S and Hoppe, AD (2013) Isolation of a novel swine influenza virus from Oklahoma in 2011

which is distantly related to human influenza C viruses. *PLoS pathog* **9**: e1003176.

Herrero, S, Millan-Leiva, A, Coll, S, Gonzalez-Martinez, RM, Parenti, S and Gonzalez-Cabrera, J (2019) Identification of new viral variants specific to the honey bee mite Varroa destructor. *Experimental and Applied Acarology* **79**: 157-168.

Hunnicutt, LE, Hunter, WB, Cave, RD, Powell, CA and Mozoruk, JJ (2006) Genome sequence and molecular characterization of Homalodisca coagulata virus-1, a novel virus discovered in the glassy-winged sharpshooter (Hemiptera: Cicadellidae). *Virology* **350**: 67-78.

Ishikawa, K, Nagai, H, Katayama, K, Tsutsui, M, Tanabayashi, K, Takeuchi, K, Hishiyama, M, Saitoh, A, Takagi, M and Gotoh, K (1995) Comparison of the entire nucleotide and deduced amino acid sequences of the attenuated hog cholera vaccine strain GPE− and the wild-type parental strain ALD. *Archives of virology* **140**: 1385-1391.

Ito, T, Suzaki, K and Nakano, M (2013) Genetic characterization of novel putative rhabdovirus and dsRNA virus from Japanese persimmon. *Journal of General Virology* **94**: 1917-1921.

Iwahashi, F, Fujisaki, K, Kaido, M, Okuno, T and Mise, K (2005) Synthesis of infectious in vitro transcripts from Cassia yellow blotch bromovirus cDNA clones and a reassortment analysis with other bromoviruses in protoplasts. *Archives of virology* **150**: 1301-1314.

Jelkmann, W (1994) Nucleotide sequences of apple stem pitting virus and of the coat protein gene of a similar virus from pear associated with vein yellows disease and their relationship with potex-and carlaviruses. *Journal of General Virology* **75**: 1535-1542.

Johnson, KN and Christian, PD (1998) The novel genome organization of the insect picorna-like virus Drosophila C virus suggests this virus belongs to a previously undescribed virus family. *Journal of General Virology* **79**: 191-203.

Joshi, S, Neeleman, L, Pleij, C, Haenni, A, Chapeville, F, Bosch, L and van Vloten-Doting, L (1984) Nonstructural alfalfa mosaic virus RNA-coded proteins present in tobacco leaf tissue. *Virology* **139**: 231-242.

Jover-Gil, S, Beeri, A, Fresnillo, P, Samach, A and Candela, H (2018) Complete genome sequence of a novel virus, classifiable within the Potyviridae family, which infects passion fruit (Passiflora edulis). *Archives of virology* **163**: 3191-3194.

Kawakami, K, Kurnia, YW, Fujita, R, Ito, T, Isawa, H, Asano, S-i, Binh, ND and Bando, H (2016) Characterization of a novel negevirus isolated from Aedes larvae collected in a subarctic region of Japan. *Archives of virology* **161**: 801-809.

Kemdirim, S, Palefsky, J and Briedis, DJ (1986) Influenza B virus PB1 protein: nucleotide sequence of the genome RNA segment predicts a high degree of structural homology with the corresponding influenza A virus polymerase protein. *Virology* **152**: 126-135.

Kuzmin, IV, Wu, X, Tordo, N and Rupprecht, CE (2008) Complete genomes of Aravan, Khujand, Irkut and West Caucasian bat viruses, with special attention to the polymerase gene and non-coding regions. *Virus Res* **136**: 81-90.

Lüthi, MN, Vorburger, C and Dennis, AB (2020) A novel RNA virus in the parasitoid wasp Lysiphlebus fabarum: genomic structure, prevalence, and transmission. *Viruses* **12**: 59.

Lanzi, G, De Miranda, JR, Boniotti, MB, Cameron, CE, Lavazza, A, Capucci, L, Camazine, SM and Rossi, C (2006) Molecular and biological characterization of deformed wing virus of honeybees (Apis mellifera L.). *Journal of virology* **80**: 4998-5009.

Le Mercier, P, Jacob, Y and Tordo, N (1997) The complete Mokola virus genome sequence:

structure of the RNA-dependent RNA polymerase. *Journal of general virology* **78**: 1571-1576.

Leat, N, Ball, B, Govan, V and Davison, S (2000) Analysis of the complete genome sequence of black queen-cell virus, a picorna-like virus of honey beesThe GenBank accession number of the sequence reported in this paper is AF183905. *Journal of General Virology* **81**: 2111-2119.

Li, C-X, Shi, M, Tian, J-H, Lin, X-D, Kang, Y-J, Chen, L-J, Qin, X-C, Xu, J, Holmes, EC and Zhang, Y-Z (2015) Unprecedented genomic diversity of RNA viruses in arthropods reveals the ancestry of negative-sense RNA viruses. *Elife* **4**: e05378.

Li, R, Gao, S, Fei, Z and Ling, K-S (2013) Complete genome sequence of a new tobamovirus naturally infecting tomatoes in Mexico. *Genome announcements* **1**.

Li, X, Xu, P, Yang, X, Yuan, H, Chen, L and Lu, Y (2017) The genome sequence of a novel RNA virus in Adelphocoris suturalis. *Archives of virology* **162**: 1397-1401.

Lozano, I and Morales, F (2009) Molecular characterisation of Rice stripe necrosis virus as a new species of the genus Benyvirus. *European journal of plant pathology* **124**: 673-680.

Mahar, JE, Shi, M, Hall, RN, Strive, T and Holmes, EC (2020) Comparative analysis of RNA virome composition in rabbits and associated ectoparasites. *Journal of virology* **94**.

Maori, E, Lavi, S, Mozes-Koch, R, Gantman, Y, Peretz, Y, Edelbaum, O, Tanne, E and Sela, I (2007) Isolation and characterization of Israeli acute paralysis virus, a dicistrovirus affecting honeybees in Israel: evidence for diversity due to intra-and inter-species recombination. *Journal of General Virology* **88**: 3428-3438.

Mari, J, Poulos, BT, Lightner, DV and Bonami, J-R (2002) Shrimp Taura syndrome virus: genomic characterization and similarity with members of the genus Cricket paralysis-like virusesThe GenBank accession number of the sequence reported in this paper is AF277675. *Journal of General Virology* **83**: 915-926.

Medd, NC, Fellous, S, Waldron, FM, Xuéreb, A, Nakai, M, Cross, JV and Obbard, DJ (2018) The virome of Drosophila suzukii, an invasive pest of soft fruit. *Virus evolution* **4**: vey009.

Melzer, M, Sether, D, Karasev, A, Borth, W and Hu, J (2008) Complete nucleotide sequence and genome organization of pineapple mealybug wilt-associated virus-1. *Archives of virology* **153**: 707-714.

Melzer, MJ, Sether, DM, Borth, WB and Hu, JS (2012) Characterization of a virus infecting Citrus volkameriana with citrus leprosis-like symptoms. *Phytopathology* **102**: 122-127.

Minicka, J, Zarzyńska-Nowak, A, Budzyńska, D, Borodynko-Filas, N and Hasiów-Jaroszewska, B (2020) High-Throughput Sequencing Facilitates Discovery of New Plant Viruses in Poland. *Plants* **9**: 820.

Murakami, R, Suetsugu, Y, Kobayashi, T and Nakashima, N (2013) The genome sequence and transmission of an iflavirus from the brown planthopper, Nilaparvata lugens. *Virus research* **176**: 179-187.

Muraki, Y, Washioka, H, Sugawara, K, Matsuzaki, Y, Takashita, E and Hongo, S (2004) Identification of an amino acid residue on influenza C virus M1 protein responsible for formation of the cord-like structures of the virus. *Journal of General Virology* **85**: 1885-1893.

Nakashima, N, Sasaki, J and Toriyama, S (1999) Determining the nucleotide sequence and capsid-coding region of Himetobi P virus: a member of a novel group of RNA viruses that infect insects. *Archives of virology* **144**: 2051-2058.

Neill, JD, Ridpath, JF, Fischer, N, Grundhoff, A, Postel, A and Becher, P (2014) Complete genome sequence of pronghorn virus, a pestivirus. *Genome announcements* **2**.

Neill, JD, Workman, AM, Hesse, R, Bai, J, Porter, EP, Meadors, B, Anderson, J, Bayles, DO and Falkenberg, SM (2019) Identification of BVDV2b and 2c subgenotypes in the United States: Genetic and antigenic characterization. *Virology* **528**: 19-29.

Nouri, S, Salem, N, Nigg, JC and Falk, BW (2016) Diverse array of new viral sequences identified in worldwide populations of the Asian citrus psyllid (Diaphorina citri) using viral metagenomics. *Journal of virology* **90**: 2434-2445.

Nunes, MR, Contreras-Gutierrez, MA, Guzman, H, Martins, LC, Barbirato, MF, Savit, C, Balta, V, Uribe, S, Vivero, R and Suaza, JD (2017) Genetic characterization, molecular epidemiology, and phylogenetic relationships of insect-specific viruses in the taxon Negevirus. *Virology* **504**: 152-167.

O'Brien, CA, McLean, BJ, Colmant, AM, Harrison, JJ, Hall-Mendelin, S, van den Hurk, AF, Johansen, CA, Watterson, D, Bielefeldt-Ohmann, H and Newton, ND (2017) Discovery and characterisation of Castlerea virus, a new species of Negevirus isolated in Australia. *Evolutionary Bioinformatics* **13**: 1176934317691269.

Obrępalska-Stęplowska, A, Budziszewska, M, Wieczorek, P and Czerwoniec, A (2012) Analysis of two strains of Peanut stunt virus: satRNA-associated and satRNA free. *Virus genes* **44**: 513-521.

Ongus, JR, Peters, D, Bonmatin, J-M, Bengsch, E, Vlak, JM and van Oers, MM (2004) Complete sequence of a picorna-like virus of the genus Iflavirus replicating in the mite Varroa destructor. *Journal of General Virology* **85**: 3747-3755.

Perera, OP, Snodgrass, GL, Allen, KC, Jackson, RE, Becnel, JJ, O'Leary, PF and Luttrell, RG (2012) The complete genome sequence of a single-stranded RNA virus from the tarnished plant bug, Lygus lineolaris (Palisot de Beauvois). *Journal of invertebrate pathology* **109**: 11-19.

Pettersson, JH-O, Ellstrom, P, Ling, J, Nilsson, I, Bergstrom, S, Gonzalez-Acuna, D, Olsen, B and Holmes, EC (2020) Circumpolar diversification of the Ixodes uriae tick virome. *bioRxiv*.

Phan, TG, del Valle Mendoza, J, Sadeghi, M, Altan, E, Deng, X and Delwart, E (2018) Sera of Peruvians with fever of unknown origins include viral nucleic acids from non-vertebrate hosts. *Virus genes* **54**: 33-40.

Picarelli, MAS, Forgia, M, Rivas, EB, Nerva, L, Chiapello, M, Turina, M and Colariccio, A (2019) Extreme diversity of mycoviruses present in isolates of Rhizoctonia solani AG2-2 LP from Zoysia japonica from Brazil. *Frontiers in cellular and infection microbiology* **9**: 244.

Presti, RM, Zhao, G, Beatty, WL, Mihindukulasuriya, KA, Da Rosa, APT, Popov, VL, Tesh, RB, Virgin, HW and Wang, D (2009) Quaranfil, Johnston Atoll, and Lake Chad viruses are novel members of the family Orthomyxoviridae. *Journal of virology* **83**: 11599-11606.

Quito-Avila, DF, Brannen, PM, Cline, WO, Harmon, PF and Martin, RR (2013) Genetic characterization of Blueberry necrotic ring blotch virus, a novel RNA virus with unique genetic features. *Journal of General Virology* **94**: 1426-1434.

Roberts, JM, Anderson, DL and Durr, PA (2018) Metagenomic analysis of Varroa-free Australian honey bees (Apis mellifera) shows a diverse Picornavirales virome. *Journal of General Virology* **99**: 818-826.

Roy, A, Choudhary, N, Guillermo, LM, Shao, J, Govindarajulu, A, Achor, D, Wei, G, Picton, D, Levy, L and Nakhla, M (2013) A novel virus of the genus Cilevirus causing symptoms similar to

citrus leprosis. *Phytopathology* **103**: 488-500.

Ryabov, EV (2007) A novel virus isolated from the aphid Brevicoryne brassicae with similarity to Hymenoptera picorna-like viruses. *Journal of General Virology* **88**: 2590-2595.

Šafářová, D, Candresse, T and Navrátil, M (2018) Complete genome sequence of a novel bromovirus infecting elderberry (Sambucus nigra L.) in the Czech Republic. *Archives of virology* **163**: 567-570.

Saito, M, Kiguchi, T, Kusume, T and Tamada, T (1996) Complete nucleotide sequence of the Japanese isolate S of beet necrotic yellow vein virus RNA and comparison with European isolates. *Archives of virology* **141**: 2163-2175.

Salánki, K, Balázs, E and Burgyán, J (1994) Nucleotide sequence and infectious in vitro transcripts of RNA 3 of tomato aspermy virus pepper isolate. *Virus research* **33**: 281-289.

Sameroff, S, Tokarz, R, Charles, RA, Jain, K, Oleynik, A, Che, X, Georges, K, Carrington, CV, Lipkin, WI and Oura, C (2019) Viral diversity of tick species parasitizing cattle and dogs in Trinidad and Tobago. *Scientific reports* **9**: 1-10.

Sasaki, J, Nakashima, N, Saito, H and Noda, H (1998) An insect picorna-like virus, Plautia stali intestine virus, has genes of capsid proteins in the 3′ part of the genome. *Virology* **244**: 50-58.

Shi, M, Lin, X-D, Tian, J-H, Chen, L-J, Chen, X, Li, C-X, Qin, X-C, Li, J, Cao, J-P and Eden, J-S (2016a) Redefining the invertebrate RNA virosphere. *Nature* **540**: 539-543.

Shi, M, Lin, X-D, Vasilakis, N, Tian, J-H, Li, C-X, Chen, L-J, Eastwood, G, Diao, X-N, Chen, M-H and Chen, X (2016b) Divergent viruses discovered in arthropods and vertebrates revise the evolutionary history of the Flaviviridae and related viruses. *Journal of virology* **90**: 659-669.

Smith, G, Macias-Muñoz, A and Briscoe, AD (2014) Genome sequence of a novel iflavirus from mRNA sequencing of the butterfly Heliconius erato. *Genome announcements* **2**.

Spear, A, Sisterson, MS, Yokomi, R and Stenger, DC (2010) Plant-feeding insects harbor double-stranded RNA viruses encoding a novel proline-alanine rich protein and a polymerase distantly related to that of fungal viruses. *Virology* **404**: 304-311.

Suzuki, T, Takeshima, Y, Mikamoto, T, Saeki, J-D, Kato, T, Park, EY, Kawagishi, H and Dohra, H (2015) Genome Sequence of a Novel Iflavirus from mRNA Sequencing of the Pupa of Bombyx mori Inoculated with Cordyceps militaris. *Genome announcements* **3**.

Tang, J, Harper, SJ, Wei, T and Clover, GR (2010) Characterization of hydrangea chlorotic mottle virus, a new member of the genus Carlavirus. *Archives of virology* **155**: 7-12.

Thawornwattana, Y, Dong, HT, Phiwsaiya, K, Sangsuriya, P, Senapin, S and Aiewsakun, P (2020) Tilapia lake virus (TiLV): Genomic epidemiology and its early origin. *Transboundary and Emerging Diseases*.

Thomas-Sharma, S, Wells-Hansen, L, Page, R, Kartanos, V, Saalau-Rojas, E, Lockhart, BE and McManus, PS (2018) Characterization of Blueberry shock virus, an Emerging Ilarvirus in Cranberry. *Plant disease* **102**: 91-97.

Thompson, JR, Fuchs, M and Perry, KL (2012) Genomic analysis of Grapevine leafroll associated virus-5 and related viruses. *Virus research* **163**: 19-27.

Valles, SM, Strong, CA, Dang, PM, Hunter, WB, Pereira, RM, Oi, DH, Shapiro, AM and Williams, DF (2004) A picorna-like virus from the red imported fire ant, Solenopsis invicta: initial discovery, genome sequence, and characterization. *Virology* **328**: 151-157.

Viljakainen, L, Borshagovski, A-M, Saarenpää, S, Kaitala, A and Jurvansuu, J (2020) Identification and characterisation of common glow-worm RNA viruses. *Virus genes*: 1-13.

Viljakainen, L, Holmberg, I, Abril, S and Jurvansuu, J (2018) Viruses of invasive Argentine ants from the European main supercolony: characterization, interactions and evolution. *Journal of General Virology* **99**: 1129-1140.

Walker, PJ, Tesh, RB, Guzman, H, Popov, VL, Travassos da Rosa, A, Reyna, M, Nunes, MR, De Souza, WM, Contreras-Gutierrez, MA and Patroca, S (2019) Characterization of three novel viruses from the families nyamiviridae, orthomyxoviridae, and peribunyaviridae, isolated from dead birds collected during west nile virus surveillance in Harris County, Texas. *Viruses* **11**: 927.

Warrilow, D, Watterson, D, Hall, RA, Davis, SS, Weir, R, Kurucz, N, Whelan, P, Allcock, R, Hall-Mendelin, S and O'Brien, CA (2014) A new species of mesonivirus from the Northern Territory, Australia. *PLoS One* **9**: e91103.

Weber, F, Gruber, S, Haller, O and Kochs, G (1999) PB2 polymerase subunit of Thogoto virus (Orthomyxoviridae family). *Archives of virology* **144**: 1601-1609.

Williams, SH, Che, X, Oleynik, A, Garcia, JA, Muller, D, Zabka, TS, Firth, C, Corrigan, RM, Briese, T and Jain, K (2019) Discovery of two highly divergent negative-sense RNA viruses associated with the parasitic nematode, Capillaria hepatica, in wild Mus musculus from New York City. *Journal of General Virology* **100**: 1350-1362.

Wilson, JE, Powell, MJ, Hoover, SE and Sarnow, P (2000) Naturally occurring dicistronic cricket paralysis virus RNA is regulated by two internal ribosome entry sites. *Molecular and cellular biology* **20**: 4990-4999.

Xing, F, Robe, BL, Zhang, Z, Wang, H and Li, S (2018) Genomic analysis, sequence diversity, and occurrence of Apple necrotic mosaic virus, a novel ilarvirus associated with Mosaic disease of apple trees in China. *Plant disease* **102**: 1841-1847.

Xu, P, Li, H, Liu, J, Luan, Y, Yin, Y and Bai, J (2011) Sequence and structure prediction of RNA-dependent RNA polymerase of lily symptomless virus isolated from L.×'Casablanca'. *Archives of virology* **156**: 939.

Yinda, CK, Ghogomu, SM, Conceição-Neto, N, Beller, L, Deboutte, W, Vanhulle, E, Maes, P, Van Ranst, M and Matthijnssens, J (2018) Cameroonian fruit bats harbor divergent viruses, including rotavirus H, bastroviruses, and picobirnaviruses using an alternative genetic code. *Virus evolution* **4**: vey008.

Zavriev, S, Kanyuka, K and Levay, K (1991) The genome organization of potato virus M RNA. *Journal of General Virology* **72**: 9-14.

Zhou, K, Yue, H, Tang, C, Ruan, W, Zhou, Q and Zhang, B (2019) Prevalence and genome characteristics of atypical porcine pestivirus in southwest China. *Journal of General Virology* **100**: 84-88.

Ziebuhr, J, Snijder, EJ and Gorbalenya, AE (2000) Virus-encoded proteinases and proteolytic processing in the Nidovirales. *Journal of General Virology* **81**: 853-879.
