# Supplementary material for: Diversity and infectivity of the RNA virome among different cryptic species of an agriculturally important insect vector: whitefly Bemisia tabaci
Source: NPJ Biofilms Microbiomes. 2021 May 13;7:43. doi: 10.1038/s41522-021-00216-5 (PMC8119434; doi:10.1038/s41522-021-00216-5)
Supplement: Supplementary file 2 — Reporting Summary [file 41522_2021_216_MOESM2_ESM.pdf]

nature research

Corresponding author(s): Jian-Ping Chen; Chuan-Xi Zhang; Jun-Min Li

Last updated by author(s): Apr 13, 2021

Reporting Summary

Nature Research wishes to improve the reproducibility of the work that we publish. This form provides structure for consistency and transparency in reporting. For further information on Nature Research policies, see our Editorial Policies and the Editorial Policy Checklist.

Statistics

For all statistical analyses, confirm that the following items are present in the figure legend, table legend, main text, or Methods section.

n/a	Confirmed	
☐	☒	The exact sample size (*n*) for each experimental group/condition, given as a discrete number and unit of measurement
☐	☒	A statement on whether measurements were taken from distinct samples or whether the same sample was measured repeatedly
☐	☒	The statistical test(s) used AND whether they are one- or two-sided<br>*Only common tests should be described solely by name; describe more complex techniques in the Methods section.*
☒	☐	A description of all covariates tested
☐	☒	A description of any assumptions or corrections, such as tests of normality and adjustment for multiple comparisons
☐	☒	A full description of the statistical parameters including central tendency (e.g. means) or other basic estimates (e.g. regression coefficient) AND variation (e.g. standard deviation) or associated estimates of uncertainty (e.g. confidence intervals)
☐	☒	For null hypothesis testing, the test statistic (e.g. *F*, *t*, *r*) with confidence intervals, effect sizes, degrees of freedom and *P* value noted<br>*Give P values as exact values whenever suitable.*
☐	☒	For Bayesian analysis, information on the choice of priors and Markov chain Monte Carlo settings
☐	☒	For hierarchical and complex designs, identification of the appropriate level for tests and full reporting of outcomes
☒	☐	Estimates of effect sizes (e.g. Cohen's *d*, Pearson's *r*), indicating how they were calculated

Our web collection on statistics for biologists contains articles on many of the points above.

Software and code

Policy information about availability of computer code

Data collection	no software was used.
Data analysis	Trinity version 2.8.5; SPAdes version 3.13.0; BWA version 0.7.17; MAFFT version 7; ModelTest-NG version 0.1.6; Bowtie2 version 2.3.5.1.

For manuscripts utilizing custom algorithms or software that are central to the research but not yet described in published literature, software must be made available to editors and reviewers. We strongly encourage code deposition in a community repository (e.g. GitHub). See the Nature Research guidelines for submitting code & software for further information.

Data

Policy information about availability of data

All manuscripts must include a data availability statement. This statement should provide the following information, where applicable:

- Accession codes, unique identifiers, or web links for publicly available datasets
- A list of figures that have associated raw data
- A description of any restrictions on data availability

All data generated in this study have been deposited in NCBI SRA/GenBank. Transcriptome (NBU-B: SRR13050950; NBU-Q: SRR13052369; FY-Q: SRR13039280;). sRNA (NBU-B: SRR13050947; NBU-Q: SRR13050948; FY-Q: SRR13082984). Newly identified viral contigs (MW256664-MW256706 and MW227222- MW227223).

Field-specific reporting

Please select the one below that is the best fit for your research. If you are not sure, read the appropriate sections before making your selection.

☒ Life sciences ☐ Behavioural & social sciences ☐ Ecological, evolutionary & environmental sciences

For a reference copy of the document with all sections, see nature.com/documents/nr-reporting-summary-flat.pdf

Life sciences study design

All studies must disclose on these points even when the disclosure is negative.

Sample size	For cross-injection experiment, a pool of 20-30 whiteflies were collected for MEAM1 and MED at each time point, and three independent biological replicates were performed.
Data exclusions	Data were not excluded from analysis.
Replication	Three independent biological replicates were performed for cross-injection experiment.
Randomization	Whiteflies used for cross-injection experiment were selected randomly.
Blinding	Blinding is not typically used in the field. Blinding during collection was not needed because conditions were well controlled.

Reporting for specific materials, systems and methods

We require information from authors about some types of materials, experimental systems and methods used in many studies. Here, indicate whether each material, system or method listed is relevant to your study. If you are not sure if a list item applies to your research, read the appropriate section before selecting a response.

Materials & experimental systems

n/a	Involved in the study	
☒	☐	Antibodies
☒	☐	Eukaryotic cell lines
☒	☐	Palaeontology and archaeology
☐	☒	Animals and other organisms
☒	☐	Human research participants
☒	☐	Clinical data
☒	☐	Dual use research of concern

Methods

n/a	Involved in the study	
☒	☐	ChIP-seq
☒	☐	Flow cytometry
☒	☐	MRI-based neuroimaging

Animals and other organisms

Policy information about studies involving animals; ARRIVE guidelines recommended for reporting animal research

Laboratory animals	This study did not involve laboratory animals.
Wild animals	This study did not involve wild animals.
Field-collected samples	The MED whitefly was originally collected from soybean plants in Suzhou (An'hui province, China) in June 2019. The whitefly culture were reared separately in insect-proof cages on cotton plants (Gossypium hirsutum L. cv. Zhemian 1793) at 25 ± 1℃, 50-70% relative humidity, and 14 h light/10 h darkness.
Ethics oversight	No ethical approval or guidance was required because whitefly is an agricultural pest.

Note that full information on the approval of the study protocol must also be provided in the manuscript.
